# Whole‐brain computational modeling reveals disruption of microscale brain dynamics in HIV infected individuals

**DOI:** 10.1002/hbm.25207

**Published:** 2020-09-17

**Authors:** Yuchuan Zhuang, Zhengwu Zhang, Madalina Tivarus, Xing Qiu, Jianhui Zhong, Giovanni Schifitto

**Affiliations:** ^1^ Department of Electrical and Computer Engineering University of Rochester Rochester New York USA; ^2^ Department of Biostatistics and Computational Biology University of Rochester Medical Center Rochester New York USA; ^3^ Department of Neuroscience University of Rochester Medical Center Rochester New York USA; ^4^ Department of Imaging Sciences University of Rochester Medical Center Rochester New York USA; ^5^ Department of Biomedical Engineering University of Rochester Rochester New York USA; ^6^ Department of Neurology University of Rochester Medical Center Rochester New York USA

**Keywords:** graph theoretical analysis, HIV‐infection, resting‐state functional MRI, tractography, whole‐brain computational modeling

## Abstract

MRI‐based neuroimaging techniques have been used to investigate brain injury associated with HIV‐infection. Whole‐brain cortical mean‐field dynamic modeling provides a way to integrate structural and functional imaging outcomes, allowing investigation of microscale brain dynamics. In this study, we adopted the relaxed mean‐field dynamic modeling to investigate structural and functional connectivity in 42 HIV‐infected subjects before and after 12‐week of combination antiretroviral therapy (cART) and compared them with 46 age‐matched healthy subjects. Microscale brain dynamics were modeled by a set of parameters including two region‐specific microscale brain properties, recurrent connection strengths, and subcortical inputs. We also analyzed the relationship between the model parameters (i.e., the recurrent connection and subcortical inputs) and functional network topological characterizations, including smallworldness, clustering coefficient, and network efficiency. The results show that untreated HIV‐infected individuals have disrupted local brain dynamics that in part correlate with network topological measurements. Notably, after 12 weeks of cART, both the microscale brain dynamics and the network topological measurements improved and were closer to those in the healthy brain. This was also associated with improved cognitive performance, suggesting that improvement in local brain dynamics translates into clinical improvement.

## Graphical abstract

In this study, we adopted the relaxed mean‐field dynamic modeling to investigate structural and functional connectivity in 42 HIV‐infected subjects before and after 12 week of combination antiretroviral therapy (cART) and compared them with 46 age‐matched healthy subjects. The results show that untreated HIV‐infected individuals have disrupted local brain dynamics that in part correlate with network topological measurements. Notably, after 12 weeks of cART, both the microscale brain dynamics and the network topological measurements improved and were closer to those in the healthy brain. This was also associated with improved cognitive performance, suggesting that improvement in local brain dynamics translates into clinical improvement.

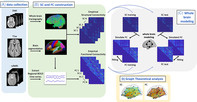

**Abbreviations**
ALFF: amplitude of low frequency fluctuations
ANI: asymptomatic neurocognitive impairment
ART: antiretroviral therapy
BBR: boundary‐based registration
cART: combination antiretroviral therapy
DCM: dynamic causal modeling
DMN: default mode network
DOF: degree of freedom
DTI: diffusion tensor imaging
DWI: diffusion weighted imaging
EPI: echo‐planar imaging
FA: fractional anisotropy
FC: functional connectivity
FDR: false discovery rate
FWHM: full width at half maximum
HAD: HIV‐associated dementia
HAND: HIV‐associated neurocognitive disorders
HC: healthy controls
HIV + 12 wk: HIV infected after 12 weeks of cART treatment
HIV + BSL: HIV infected treatment‐naïve patients at baseline
MD: mean diffusivity
MFM: mean‐filed dynamic modeling
MND: mild neurocognitive disorder
MPRAGE: magnetization‐prepared rapid acquisition gradient echo
PCC: posterior cingulate cortex
PFT: particle filtering tractography
PSC: population‐based structural connectome
PVE: partial volume estimation
rMFM: relaxed mean‐field dynamic modeling
SC: structural connectivity
T1w: T1‐weighted
TE: echo time
TI: inversion time
TR: repetition time
WHO: World Health Organization

## 1 INTRODUCTION

There were approximately 37.9 million people globally living with the HIV in 2018 according to World Health Organization (WHO, https://www.who.int/gho/hiv/en/). Since the introduction of antiretroviral therapy (ART), there has been a significant decrease in the mortality rate of people infected with HIV, and a dramatic decrease in the incidence of HIV‐associated dementia (Saylor et al., 2016). However, the prevalence of HIV‐associated neurocognitive disorders (HAND) is increasing (Nabha, Duong, & Timpone, 2013). The cause of the high prevalence of HAND remains unclear. It is likely multifactorial, including early injury prior to starting antiretroviral drugs (HIV infects the brain shortly after transmission), chronic mild neuroinflammation and possibly neurotoxicity of antiretroviral drugs (Chang & Shukla, 2018; Fois & Brew, 2015).

MRI‐based neuroimaging techniques are valuable in the investigation of HIV‐infection associated neuropathology (Chang & Shukla, 2018). HIV‐infected subjects have reduced cortical thickness and subcortical brain volumes (Chang & Shukla, 2018; Sanford, Fellows, Ances, & Collins, 2018), and reduced functional connectivity (FC) (Samboju et al., 2018; Zhuang et al., 2017). Both imaging metrics have been associated with impaired cognitive performance when compared with healthy controls (HCs) (Samboju et al., 2018; Sanford et al., 2018; Zhuang et al., 2017). Brain injury at a microstructural level can be quantified by diffusion tensor imaging (DTI) metrics, such as decreased fractional anisotropy (FA) and increased mean diffusivity (MD). Both FA and MD abnormalities have been previously reported in HIV‐infected subjects (Stebbins et al., 2007; Underwood et al., 2017; Zhu et al., 2013). Graph theoretical analysis of structural and functional connectome has also been used to show brain network topological changes in HIV‐infection (Abidin et al., 2018; Bell et al., 2018; Chockanathan, AM, Abidin, Schifitto, & Wismuller, 2018; Thomas, Brier, Ortega, Benzinger, & Ances, 2015).

Overall, there is evidence that brain injury is present and measurable via MRI in HIV infected individuals. However, most of the previous studies have investigated one modality at a time, whereas the integration of multimodalities in HIV‐related studies has not been fully explored yet. Large‐scale whole‐brain dynamic modeling is a promising approach to further quantify the integrated contribution of structural and FC in CNS injury. This approach allows simulating resting‐state fluctuations emerging from the interaction between brain regions, constrained by the anatomical connections derived from DTI, and effectively integrates FC and structural connectivity (SC) (Honey et al., 2009; Mollink et al., 2019; Surampudi et al., 2019; Wang et al., 2019). Furthermore, this approach has already provided some insights in connectome disruption in other neurological disorders such as Alzheimer's disease (AD) and Parkinson's disease (Alderson et al., 2018; Deco & Kringelbach, 2014; Demirtas et al., 2017; Jirsa, Sporns, Breakspear, Deco, & McIntosh, 2010; Proix, Bartolomei, Guye, & Jirsa, 2017). A novel whole‐brain modeling technique, named relaxed mean field dynamic modeling (rMFM) (Wang et al., 2019) has been proposed to simulate local brain dynamics. Previous whole‐brain modeling studies assumed that local microscale properties, the recurrent connection strengths and subcortical inputs, were the same across entire brain, while the rMFM modeling method relaxed these two parameters to be heterogeneous across different brain regions. Two microscale brain properties, recurrent connection strengths and subcortical inputs, can be derived from this generative brain dynamic modeling by tuning the model to fit the simulated FC to empirical FC.

In this study, we applied the rMFM (Wang et al., 2019) to assess CNS changes in a cohort of HIV infected treatment‐naïve patients at baseline (HIV + BSL) and after 12 weeks of combination antiretroviral therapy (cART) treatment (HIV + 12 wk), and compared them to HCs. The overreaching goal was to investigate potentially new imaging biomarkers that could be sensitive to CNS changes and thus helpful in monitoring CNS disease progression and response to treatment. First, we built the rMFM whole‐brain dynamic models for the three groups (HC, HIV + BSL, HIV + 12 wk), respectively. We then compared the microscale brain properties (the recurrent connection strength and subcortical inputs) across the three groups. This was followed by investigating the topological changes among the three groups using traditional graph theoretical analysis from both global and regional perspectives. Subsequently, we investigated the association of nodal graph theoretical measurements with microscale brain properties and neuropsychological tests scores.

## 2 MATERIALS AND METHODS

### 2.1 MRI data collection

MRI was performed on a 3 T Siemens MAGNETOM Trio MRI scanner (Siemens Healthineer, Germany) equipped with a 32‐channel head coil. T1‐weighted three‐dimensional magnetization‐prepared rapid acquisition gradient echo images were acquired, with repetition time (TR)/inversion time (TI)/echo time (TE) = 2,530/1,100/3.44 ms, voxel size = 1.0 × 1.0 × 1.0 mm^3^, flip angle = 7°, bandwidth = 190 Hz/pixel. Diffusion weighted imaging (DWI) data were acquired with the following parameters: TR/TE = 34.85/7.12 ms; 10 *b* = 0 s/mm^2^ images; 60 diffusion weighting images with *b* = 1,000 s/mm^2^ and direction uniformly distributed on a unit sphere; voxel size = 2 × 2 × 2 mm^3^, bandwidth = 1,502 Hz/pixel. A double‐echo gradient echo field map sequence was acquired with the same resolution as the DWI sequence and was used to correct for distortion caused by B0 inhomogeneity. Resting‐state fMRI data were acquired using a gradient echo‐planar imaging sequence, with TR/TE = 2,000/30 ms, voxel size = 4 × 4 × 4 mm^3^, 150 time points, flip angle = 90°, bandwidth = 1,562 Hz/pixel. During the entire 5‐min resting‐state fMRI series, participants were instructed to keep their eyes closed and avoid falling asleep.

### 2.2 Overview of data processing

**FIGURE 1:**
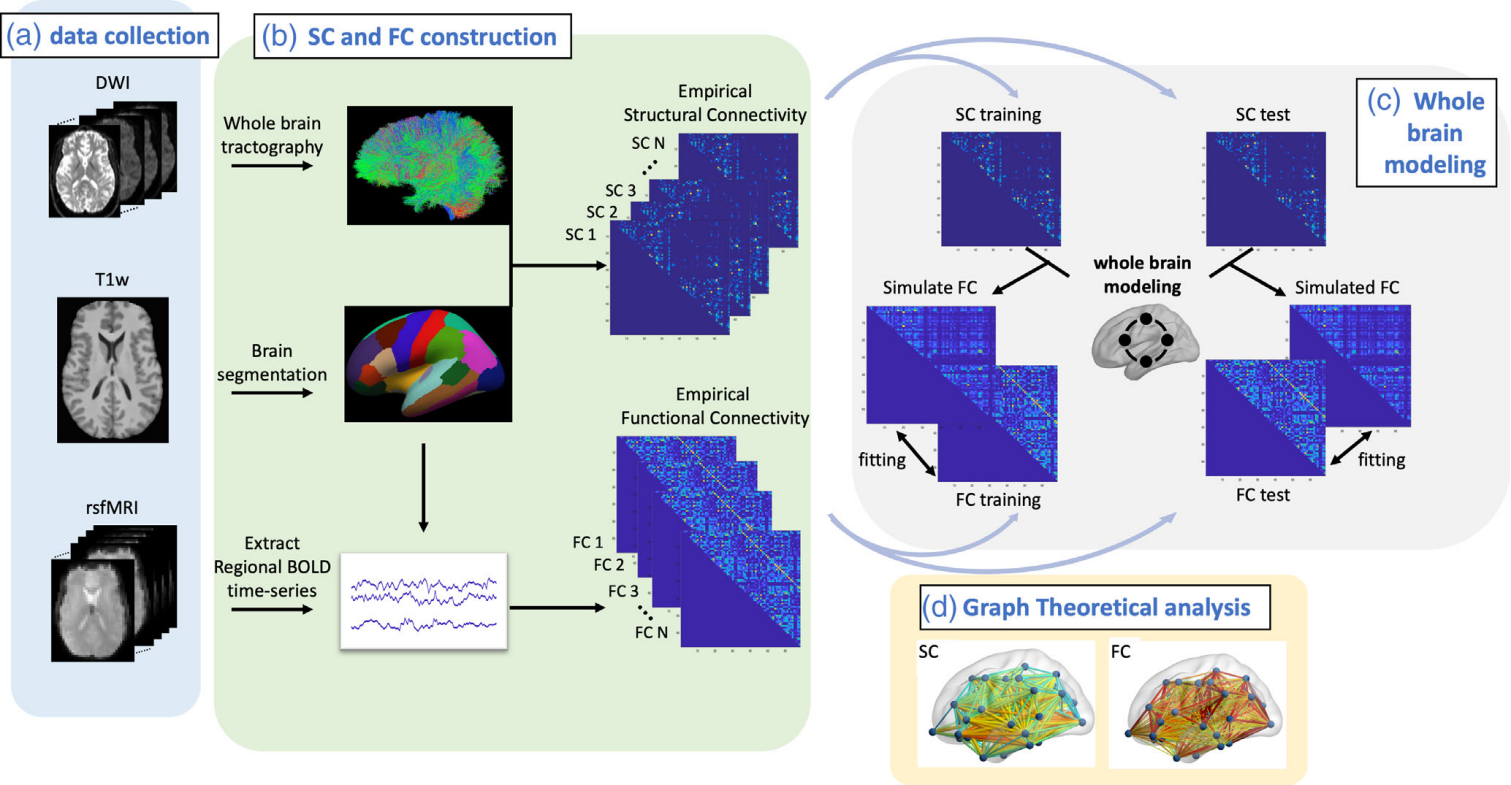
Overview of processing pipeline. (a) We collected diffusion weighted image (DWI), T1‐weighted (T1w) structural image, and resting‐state functional MRI (rsfMRI) for each HIV+ and healthy control (HC) subject. (b) For each subject, the T1w image was segmented using Freesurfer. DWI and rsfMRI were preprocessed and coregistered to T1w image. Whole‐brain tractography for each subject was derived from DWI image, and a structural connectivity (SC) matrix was generated for each subject. The streamline count for each pair of brain regions constitutes the SC matrix. Regional BOLD time series were extracted for each subject. The Pearson's correlation coefficient of the BOLD time series of two brain regions constitutes the functional connectivity (FC) matrix. (c) Whole‐brain dynamic modeling, specifically, relaxed mean field dynamic modeling (rMFM), is used to study the neuronal dynamic changes between HIV+ subjects and HC subjects. (d) We also used graph theoretical analysis to investigate the topological changes by comparing the SC and FC for HIV+ subjects with HC subjects

The processing pipeline schematically shown in Figure 1 has the following steps:

1. We preprocessed the T1‐weighted (T1w) image, DWI, and rsfMRI, and constructed the empirical SC and FC (Section 2.3).
2. The rMFM whole‐brain model was built for each group (Section 2.4): We split the empirical SC and FC into training and test datasets for each cohort. We then derived the rMFM model parameters using the training dataset and validated each rMFM model using the test datasets (Section 2.5). The microscale brain properties, namely recurrent connection strength, denoted as *w*, and subcortical input strength, denoted as *I*, were derived from each group were then compared among HIV + BSL, HIV + 12 wk and HC subjects.
3. Graph topological analysis on empirical FC and SC (Section 2.6): We investigated the topological changes in empirical FC and correlated the recurrent connection strength *w*, and subcortical inputs *I* with the nodal graph theoretical measurements.
4. The rMFM modeling and graph theoretical analysis were validated in three steps (see supplementary material): (a) split the data into different training and testing groups, (b) doubled the rMFM optimization steps, and (c) reproduced our results using a finer segmented brain atlas, Destrieux atlas (Destrieux, Fischl, Dale, & Halgren, 2010), which includes 148 cortical regions.
5. Neuropsychological assessment scores were compared among cohorts, and their relations with graph theoretical measurements were also investigated (Section 2.7).

### 2.3 MRI data preprocessing

#### 2.3.1 Anatomical T1w image analysis

Three‐dimensional high resolution T1w images were brain extracted using Brain Extraction Tool (Smith, 2002) to remove nonbrain tissue. Cortical and subcortical segmentation were then performed with Freesurfer image analysis suite (http://surfer.nmr.mgh.harvard.edu/). The Desikan–Killiany atlas (Desikan et al., 2006) and the Destrieux atlas (Destrieux et al., 2010) were mapped to individual T1w images and were further used for SC and FC construction. The 68 labeled cortical regions, 34 in each hemisphere from the Desikan–Killiany atlas are served as nodes in the SC and FC. The Destrieux atlas includes 148 labeled cortical regions, 74 in each hemisphere. The main results in the following sections are calculated using Desikan–Killiany atlas, whereas the Destrieux atlas was used for validation of rMFM modeling results (supplementary materials).

#### 2.3.2 DTI data processing and whole‐brain tractography

Preprocessing of DTI data was reported in detail previously (Zhuang et al., 2017). Briefly, the b0 images and diffusion‐weighted images were motion corrected using a 6‐degrees of freedom rigid‐body registration and field maps were used to correct the susceptibility induced distortion, using FUGUE in FSL (Jenkinson, 2003). DTI processing and structural connectome construction were then performed using the population‐based structural connectome pipeline (Zhang et al., 2018). A reproducible probabilistic whole‐brain tractography algorithm (Girard, Whittingstall, Deriche, & Descoteaux, 2014; Maier‐Hein et al., 2017) was used to reconstruct streamlines. The tissue partial volume estimation maps obtained from the anatomical T1w image helped reduce the tractography bias. The particle filtering tractography was used to reconstruct more reliable streamlines (Girard et al., 2014). After whole‐brain tractography was generated, the Desikan–Killiany atlas and Destrieux atlas were used to construct the structural connectome represented by 68 × 68 matrix and 148 × 148 matrix, respectively. The individual SC matrix was used for graphic theoretical analysis. Subjects were randomly split into training and test groups, controlling for age, so that there was no significant age difference between the training and testing group, and between the three cohorts (see Supplemental Table S1). For the whole‐brain dynamic modeling, the SC matrices were averaged across subjects in training and test datasets, respectively.

#### 2.3.3 Resting‐state functional MRI analysis

Resting‐state functional MRI data were preprocessed using FMRI Expert Analysis Tool (Woolrich, Ripley, Brady, & Smith, 2001), part of FSL (Jenkinson, Beckmann, Behrens, Woolrich, & Smith, 2012). Detailed preprocessed steps are included in the supplementary materials. The preprocessed fMRI data were then further cleaned by using FIX (Griffanti et al., 2014; Salimi‐Khorshidi et al., 2014), an ICA‐based noise removal software that automatically classifies noise components of the resting‐state functional data. Head motion, white matter, and cerebrospinal fluid time series were regressed out as nuisance regressors. Two HIV + BSL subjects and one HC subject were removed from rsfMRI analysis due to head motion greater than 2.5 mm. We used a two‐sample *t* test to ensure there was no significant head motion difference between HIV+ and HC cohort (HIV+ mean displacement = 0.748 mm, HC mean displacement = 0.645 mm, *p* = .065).

Mean BOLD signals were then extracted from each parcel in the Desikan–Killiany and Destrieux atlases. Pearson's correlation of the mean BOLD signals between each pair of parcels was calculated yielding an FC matrix for each subject. The FC matrix was then Fisher's *r*‐to‐*z* transformed, resulting in a zFC matrix. The zFC matrices for all subjects were used for graph theoretical analysis. In preparation for rMFM whole‐brain dynamic modeling, the zFC matrices were averaged across subjects within training or test dataset, for each age‐controlled cohort (HIV + BSL, HIV + 12 wk, and HC) respectively (Supplemental Table S1).

### 2.4 Simulated FC using rMFM model

The whole‐brain dynamic mean‐field model (MFM) (Deco et al., 2013; Deco et al., 2014) has been widely used to derive spontaneous brain activity from SC (Deco et al., 2018). Here, we used a modified version of MFM, the rMFM (Wang et al., 2019), which assumes the recurrent connection strengths *w* and subcortical inputs *I* are not uniformly distributed in the brain. This model has been previously proved to improve the FC simulation by 53% over the original MFM (Wang et al., 2019). Using rMFM, we simulated the neural activity for each cortical region, and then used the Balloon–Windkessel hemodynamic model (Buxton, Wong, & Frank, 1998; Friston, Harrison, & Penny, 2003; Friston, Mechelli, Turner, & Price, 2000) to convert neural activity to simulated BOLD signal. The simulated FC was then calculated using Pearson's correlation of the BOLD signal for pairwise cortical regions. More details of the rMFM model and its mathematical relations to various parameters are given in supplementary materials.

### 2.5 Model parameters estimation

Empirical SC and FC for each group (HIV + BSL, HIV + 12 wk, and HC) in the training dataset was used to estimate the model parameters. There are 138 parameters to be optimized when we use the Desikan atlas (68 recurrent connection strength *w*_i_, 68 subcortical input strength *I*_i_, global scaling factor *G*, and noise coefficient σ).

The optimization steps were based on the expectation–maximization algorithm in dynamic causal modeling (Friston et al., 2003), and were detailed in (Wang et al., 2019). In this study, 500 iterations were performed for each run, and the final optimum estimated parameters for the rMFM model were chosen from the highest Pearson's correlation coefficient between the empirical FC and simulated FC in the training dataset from the 500 iterations (highest similarity for each cohort in Supplemental Figure S1a). The corresponding 138 model parameters were stored, shown as one column in Supplemental Figure S2.

We noticed that the random initialization parameters had a small effect on the final model parameters, so we repeated the entire optimization process with 25 different random initializations for each cohort. We then chose the top five sets of model parameters, that is, the model parameters corresponding to the five highest Pearson's correlation coefficients between empirical FC and simulated FC (the five highest similarities in Supplemental Figure S1b).

We then fed the empirical SC in the test dataset for each group to its own rMFM model, to validate these three rMFM models by calculating the similarity between the simulated FC and empirical FC for each group. After each model for HIV + BSL, for HIV + 12 wk, and for HC was validated, we obtained three rMFM generative models representing the three cohorts. The recurrent connection *w* = {*w*_1_, …, *w*_*n*_} and subcortical inputs *I* = {*I*_1_, …, *I*_*n*_}, where *n* = 68 for Desikan–Killiany atlas, in each rMFM model represent the microscale brain dynamics for each group and were compared among groups.

To ensure 500 iterations for each optimization run were sufficient, we also repeated the process with 1,000 iterations, with 15 different random initializations for each group. In addition, we repeated the rMFM modeling process using a different atlas to validate our results. To do this, we constructed the empirical SC and FC using Destrieux atlas, which yields 298 model parameters to be optimized. Detailed results from these analyses are reported in the supplementary materials.

### 2.6 Graph theoretical analysis

The topology of the empirical FC and SC was further evaluated using graph theoretical measurements (Rubinov & Sporns, 2010), with calculations using Brain Connectivity Toolbox (BCT, https://sites.google.com/site/bctnet/) and graph theoretical network analysis toolbox (GRETNA (Wang et al., 2015), https://www.nitrc.org/projects/gretna).

It is known that there are some spurious connections in the connectivity matrix that should be taken into consideration in the analysis. Also, applying arbitrary thresholding of the FC or SC metrics before calculating the topology properties may influence the result (van Wijk, Stam, & Daffertshofer, 2010). Therefore, we applied a range of thresholds to study the topology properties under different network sparsity. The graph theoretical measurements were calculated over 10 different network sparsity values ranging from 0.05 to 0.5, where the network sparsity is defined as the ratio of the number of edges divided by the maximum possible number of edges in a network. Undirected weighted matrices were used in these calculations.

Four common topological measurements were chosen in this study for analysis of network properties, including the clustering coefficient, shortest path length, global efficiency, and smallworldness. Detailed information is included in supplementary materials. We evaluated the association between the nodal graph theoretical measurements and the recurrent connection strength *w*, and the subcortical inputs *I*, using Pearson's correlation.

### 2.7 Neuropsychological assessment

The neurocognitive evaluation was performed by trained staff and supervised by a neuropsychologist, and included tests of executive function (Trailmaking Test Part B, Stroop Interference Task), speed of information processing (Symbol Digit Modalities Test and Stroop Color Naming), attention and working memory (CalCAP[CRT4] and WAIS‐III Letter‐Number Sequencing), learning (Rey Auditory Verbal Learning Test RAVLT [trials 1–5], Rey Complex Figure Test Immediate Recall), memory (Rey Auditory Verbal Learning Test RAVLT Delayed Recall, Rey Complex Figure Test Delayed Recall), verbal fluency (Letter, Category and Action Fluency Tasks), and motor (Grooved Pegboard, the left and right hands). An estimate of premorbid intellectual functioning ability was obtained via WRAT‐4 Reading. The total composite Z‐score was the primary cognitive outcome and was created from the linear combination of the Z‐scores of the seven cognitive domains measured (executive function, speed of information processing, attention and working memory, learning, memory, verbal fluency, and motor). HAND diagnoses were determined for each participant according to the Frascati criteria (Antinori et al., 2007). The neuropsychological composite Z‐score and the seven cognitive domains Z‐scores were compared between HIV + BSL, HIV + 12 wk, and HC, respectively. We also investigated the relationship between the graph theoretical measurements with cognitive performance scores.

### 2.8 Statistical analysis

Comparisons of continuous variables between two independent groups were conducted by two‐group Welch's unequal variances *t* test. Fisher's exact test was used to test any proportional differences in race, gender, and education level between the HIV+ and control groups in Table 1. Pearson's correlation test was used to analyze the association between two continuous variables. A *p*‐value *p* < .05 was considered statistically significant for a single hypothesis testing problem. For inferential problems that involved multiple hypotheses, Benjamini–Hochberg multiple testing procedure was used to control the false discovery rate (FDR) at <.05 level. The statistical analysis in this study was performed using MATLAB 2017b (https://www.mathworks.com/products/matlab.html) and R version 3.6.1 (https://www.r-project.org/).

**TABLE 1:** Demographics and baseline clinical variables

	HIV infected (*n* = 42)	HC (*n* = 46)	*p*‐Value
Age (years) mean ± *SE*	34.9 ± 2.2	37.3 ± 2.1	.196
Gender (male:female)	39:3	23:23	<.001
Ethnicity (White:Black:Other)	22:19:1	35:5:6	.042
Education			
≤12 years	12	6	1
>12 years	30	40	
HIV duration by patient report at baseline (months) median (lower quantile, upper quantile)	1 (1, 12.75)	NA	—
Baseline CD4 cell count (cells/mm^3^) mean ± *SE*	515.8 ± 42.3	NA	—
Baseline HIV RNA levels (log_10_ unit) median (range)	4.5 (1.7, 5.8)	NA	—
HAND classification at baseline			
Normal	21	NA	1
ANI	20	NA	
MND	1	NA	
Total NPZ score mean (*SD*)			
BSL	−1.886 (3.79)	0.100 (3.94)	.014
12 week	−0.187 (3.88)	—	

Abbreviations: 12‐week, HIV+ after 12‐weeek cART treatment; ANI, asymptomatic neurocognitive impairment; BSL, HIV+ and HC at baseline; CD4, cluster of differentiation 4; HAND, HIV‐associated neurocognitive disorder; HC, healthy control; MND, mild neurocognitive disorder; NPZ score, neuropsychological test Z‐score; SE, standard error.

## 3 RESULTS

### 3.1 Demographics

Then, 42 HIV+ subjects were age matched with 46 HC. The HIV+ subjects were scanned before starting the cART treatment, and scanned, on average, after 12‐week of the initiation of cART treatment. At baseline, 21 HIV+ individuals had normal cognitive performance, 20 had asymptomatic neurocognitive impairment, and one had mild neurocognitive disorder. The overall cognitive performance, based on the summary Z‐score of all cognitive tests, was significantly higher in the HC compared to HIV infected individuals. The mean CD4 count and HIV RNA plasma levels at baseline were 515.8 ± 42.3 cells/mm^3^, and 4.254 ± 0.164 log_10_ copies/ml, respectively. After 12 weeks, mean CD4 cell count and HIV RNA levels were 566.4 ± 44.5 cells/mm^3^ and 2.675 ± 2.641 log_10_ copies/ml, respectively.

### 3.2 FC simulation using rMFM

We performed rMFM simulations with 25 different random initializations for each cohort, respectively, 75 simulations in total. Supplemental Figure S1a shows the increase of similarity within 500 iterations for model optimization. The similarities within each cohort are consistent, and all above 0.55, indicating that the rMFM whole‐brain dynamic model yields good simulation of FC from SC in the training dataset of each cohort. The similarity between simulated FC and empirical FC shows high consistency across different random initializations.

We found that using different initialization parameters had little effect on the final similarity results (see Supplemental Figure S1b). The variance of the maximum similarity Z‐score for 25 random initializations for HC, HIV + BSL, and HIV + 12 wk are 5.438 × 10^−5^, 8.886 × 10^−5^, and 4.210 × 10^−5^, respectively. Different initialization parameters also had little effect on the final rMFM model parameters (see Supplemental Figure S2), including the recurrent connection *w*, excitatory subcortical input *I*, global scaling of the SC *G*, and neuronal noise σ. Therefore, to minimize this effect, for each cohort, the optimum rMFM model was adopted from the averaged values across the five runs which had the highest similarities.

### 3.3 Model validation

**FIGURE 2:**
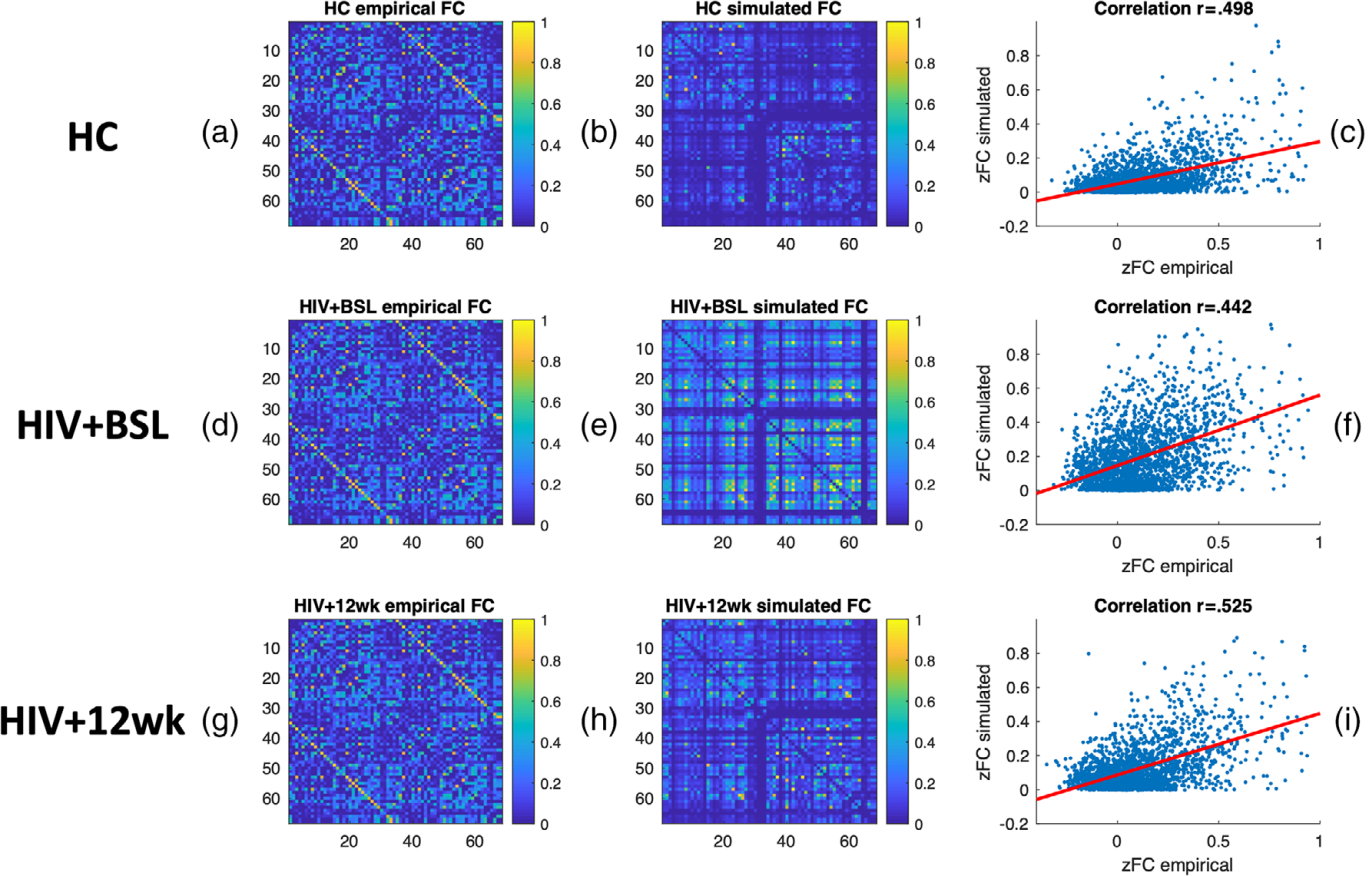
Relaxed mean field dynamic modeling (rMFM) simulated functional connectivity (FC). The empirical and rMFM models generated the simulated FC for healthy control (HC), HIV + BSL, and HIV + 12 wk, respectively. Left column (a,d,g): the averaged empirical FC in test dataset for each cohort. Middle column (b,e,h): the averaged simulated FC. Right column (c,f,i): The correlation between empirical and simulated FC

We validated the rMFM model parameters for each cohort using the test dataset. Then we calculated the simulated neuronal activity for each brain region by feeding the empirical SC from the test dataset into the fitted rMFM model for each cohort. We calculated the simulated BOLD signal for each region by feeding the simulated neuronal activity to the Balloon–Windkessel hemodynamic model. The simulated FC was calculated by Pearson's correlation. We run 1,000 simulations with different random initializations for each cohort using the test dataset. The mean simulated FC matrices for each cohort were calculated as the average across the 1,000 simulations (see Figure 2b,e,h). The correlation between the averaged simulated and empirical FC in the test dataset is 0.498, 0.442, and 0.525 for HC, HIV + BSL, and HIV + 12 wk, respectively (see Figure 2c,i,f). The empirical SC‐FC correlation coefficients in the test dataset are 0.401, 0.409, and 0.414, respectively, suggesting that the use of rMFM model for simulating FC from SC improved their agreements by 24.2, 8.1, and 26.8%.

Further validation steps including: (a) using a different training/test dataset, (b) increase to 1,000 iterations, and (c) reproducing results using Destrieux atlas, which can be found in the supplementary materials.

### 3.4 rMFM model parameters comparison between HIV and HC

**FIGURE 3:**
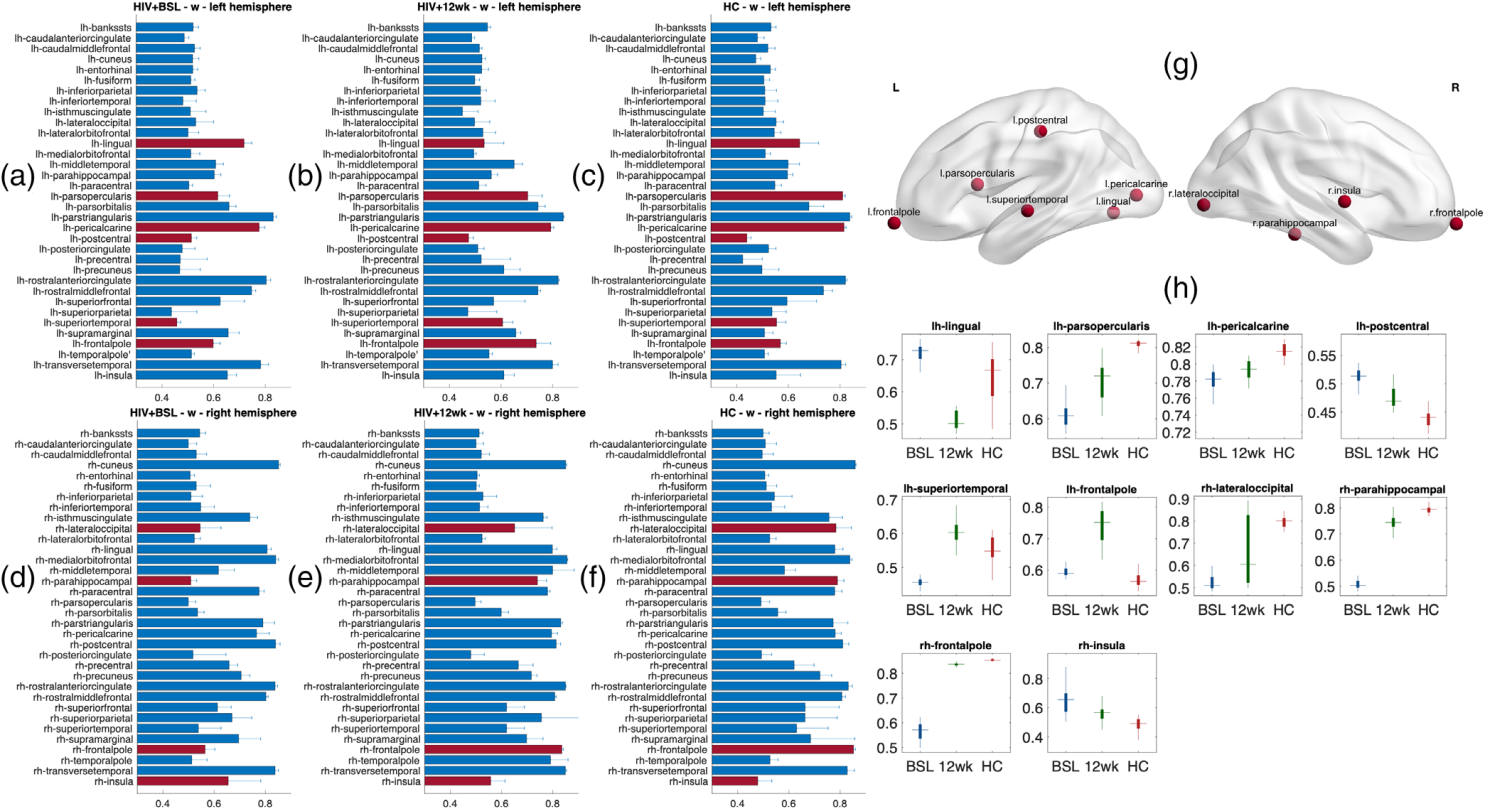
Recurrent connection strengths. (a–f) The regional recurrent connection strength (*w*_*i*_) in three groups; (a,d) HIV + BSL; (b,e) HIV + 12 wk; and (c,f) HC. (a–c)—left hemisphere; (d–f)—right hemisphere. The bars and error bars indicate the mean and *SD* of the recurrent connection strength for each brain region, the red bars indicate the regions which are significantly different between cohorts (false discovery rate [FDR] *p* < .01). (g) The regional recurrent connection changes in these nodes (red bars in figure (a–f)) plotted on a smoothed brain surface indicate their anatomical locations. (h) Bar plots show the recurrent connection strength for each ROIs. Red: HC, blue: HIV + BSL, green: HIV + 12 wk

**FIGURE 4:**
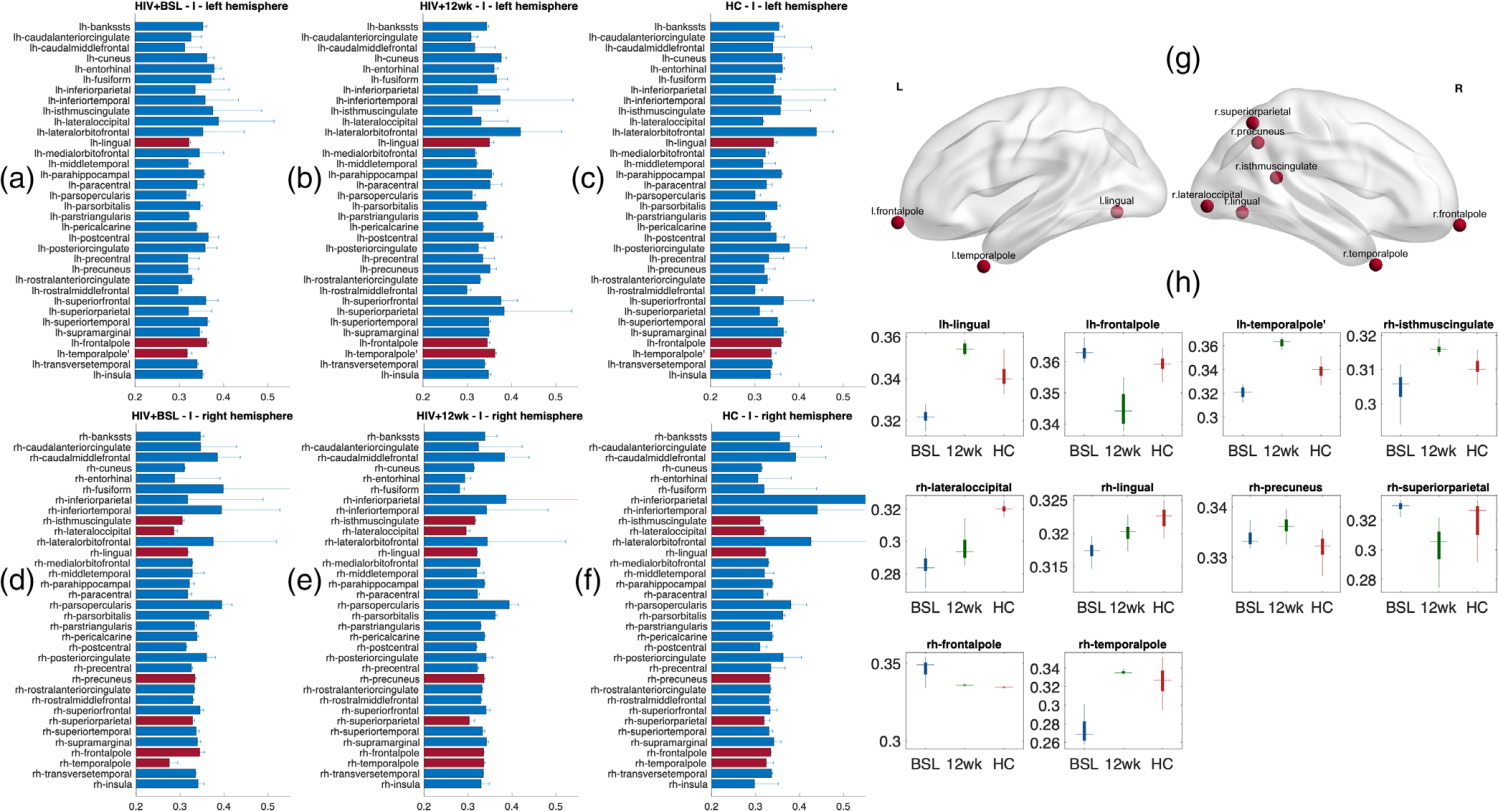
Subcortical input strengths. (a–f) The subcortical input strength (*I*_*i*_) in the three groups: (a,d) HIV + BSL; (b,e) HIV + 12 wk; and (c,f) healthy control (HC), in both hemisphere; (a–c)—left hemisphere; (d–f)—right hemisphere. The bars and error bars indicate the mean and *SD* of the subcortical input strength for each brain region, the red bars indicate the regions which are significantly different between cohort (false discovery rate [FDR] *p* < .01). (g) The subcortical inputs changes in these nodes plotted on a smoothed brain surface indicate their anatomical locations. (h) Bar plots show the subcortical input strength for each ROI. Red: HC, blue: HIV + BSL, green: HIV + 12 wk

The microscale brain dynamic properties of the three groups were investigated by comparing the rMFM model parameters, *w* and *I*. Here, we compared these two regional values between cohorts to identify which brain regions changed significantly with a treatment intervention. The recurrent connection strength *w* is shown in Figure 3, while the subcortical inputs *I* are shown in Figure 4.

The recurrent connection strengths for each region and cohort *w*_i_, are reported in Figure 3a–f. There were 10 regions that were significantly different when comparing HIV + BSL, HIV + 12 wk, and HC (shown in Figure 3 red bar, also shown in Figure 3g, FDR corrected *p* < .01). These regions are the left frontal pole, opercular part of inferior frontal gyrus (pars opercularis), superior temporal gyrus, postcentral gyrus, pericalcarine cortex, lingual gyrus; right frontal pole, insula, parahippocampal gyrus, and lateral occipital lobe. Their anatomical locations are shown in Figure 3g. The boxplots in Figure 3h show the recurrent connection strength *w* in these regions, there are 9 out of 10 regions showing clear transitions toward HCs values after 12 weeks of cART treatment.

The subcortical input strength for each region *I*_i_, and for each cohort are reported in Figure 4a–f. The red bars indicate the regions with significant differences among the three groups. The regions include the left frontal pole, temporal pole, and lingual gyrus; right frontal pole, temporal pole, superior parietal lobule, precuneus, isthmus of the cingulate gyrus, lateral occipital lobe, and lingual gyrus. Their anatomical locations are shown in Figure 4g. We also observed that 9 out of 10 brain regions showed transitions toward HC after 12 weeks of treatment, for example in left and right frontal pole and right precuneus (see Figure 4h). We also observed that the regions which differed in subcortical inputs *I* were asymmetric (see Figure 4g), with more representation in the right posterior regions. These results are also replicated when using Destrieux atlas (see Supplemental Figure S11).

### 3.5 Graph theoretical analysis on empirical FC, correlation with recurrent connection strength *w*

**FIGURE 5:**
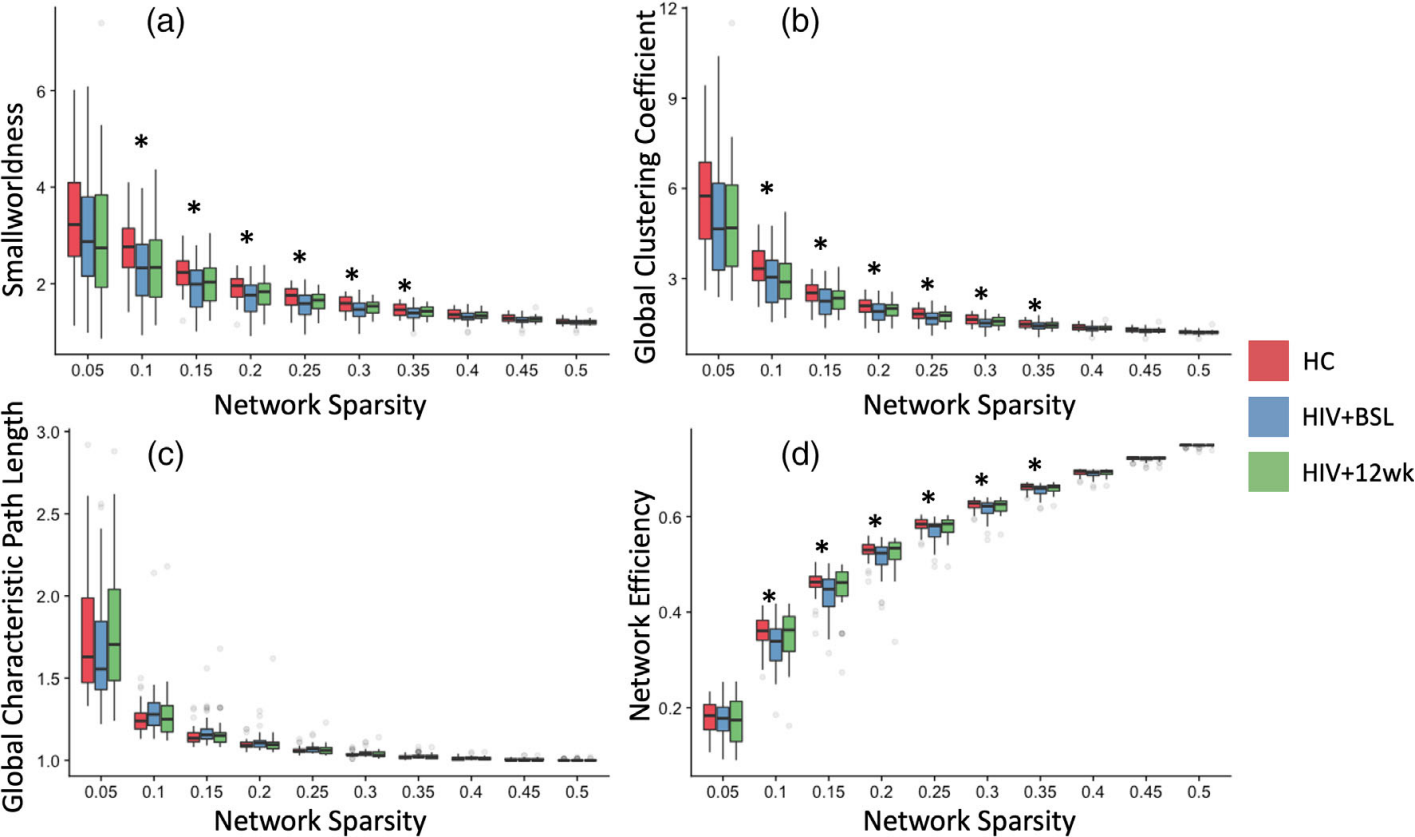
Graph theoretical measurements. Graph theoretical measurements on binarized functional connectivity matrix across network sparsity range from 0.05 to 0.5. (a) Smallworldness. (b) Global clustering coefficient. (c) Global characteristic path length. (d) Global network efficiency. *False discovery rate (FDR) corrected *p* < .05. Red: healthy control (HC), blue: HIV + BSL, green: HIV + 12 wk

We have investigated the functional network topology using the conventional graph theoretical analysis. We calculated both global and nodal clustering coefficient, network efficiency, shortest path length, and smallworldness. The global network topology differences between groups are reported in Figure 5. Using the empirical FC, we have found that, the smallworldness (Figure 5a), normalized clustering coefficient (gamma) (Figure 5b), and global efficiency (Figure 5d) were significantly reduced in HIV + BSL compared to HC, in a majority of network sparsity range (sparsity range: 0.05–0.5, FDR corrected *p* < .05). There were no significant differences between HIV + 12 wk and HC, or HIV + BSL and HIV + 12 wk after FDR correction. Here, we only report the results for the binarized FC matrix across different network sparsity. Similar results were reproduced using the weighted FC matrix (Supplemental Figure S12). We also investigated the network topology on the empirical SC but did not find any statistically significant difference between groups.

**FIGURE 6:**
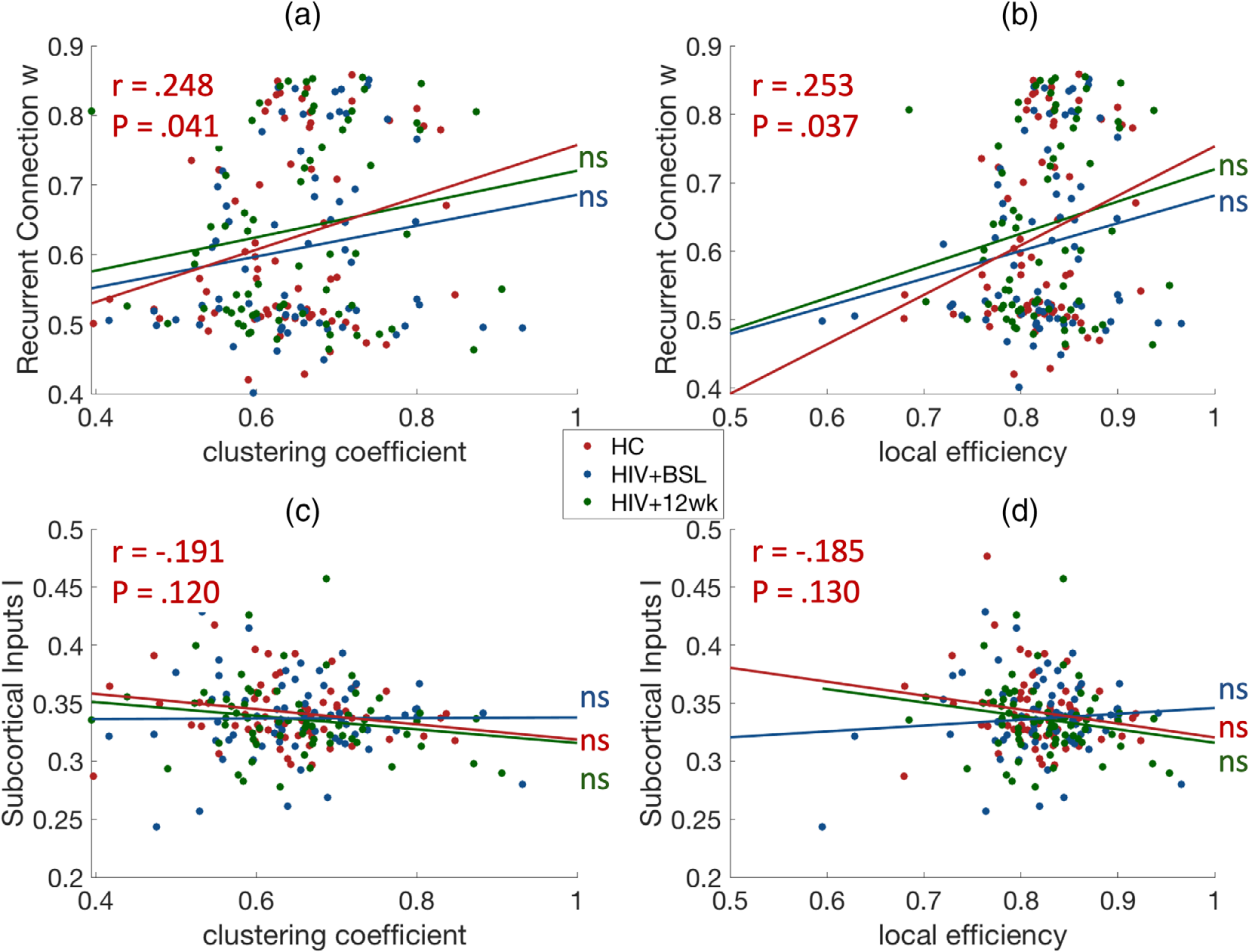
Recurrent connection correlated with network topology. The recurrent connection *w* and subcortical inputs *I* correlated with empirical local network topology. (a) Association between clustering coefficient and recurrent connection strength *w*. (b) Association between local efficiency and recurrent connection strength *w*. (c) Association between clustering coefficient and subcortical inputs *I*. (d) Association between local efficiency and subcortical inputs *I*. Red: healthy control (HC), blue: HIV + BSL, green: HIV + 12 wk. ns: not significant

Next, we investigated the association between local topological measurements and rMFM model parameters. The nodal clustering coefficient and local efficiency results were correlated with recurrent connection strengths *w* and subcortical inputs *I*, as shown in Figure 6. We found significant correlations between recurrent connection strengths *w*, nodal clustering coefficient (*r* = .248, *p* = .041), and local efficiency (*r* = .253, *p* = .037) only in the HC group. These relationships were confirmed using Destrieux atlas (Supplemental Figure S13).

### 3.6 Neuropsychological test score comparison, association with FC graph theoretical measurements

**FIGURE 7:**
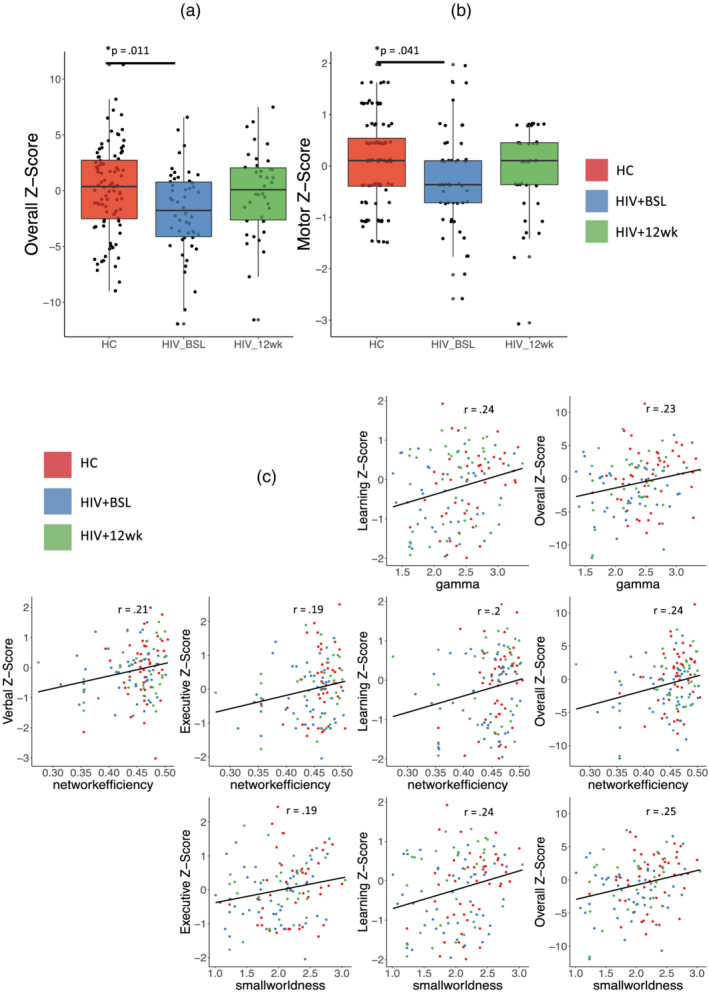
Neuropsychological test. (a) Overall composite Z‐score. (b) Motor Z‐score. (c) Linear correlation between empirical functional connectivity (FC) graph theoretical measurements and Neuropsychological test Z‐score. Red: healthy control (HC), blue: HIV + BSL, red: HIV + 12 wk. All of these plots reach to significance level: uncorrected *p* < .05. Gamma = normalized network clustering coefficient

As shown in Figure 7, HIV + BSL subjects had lower overall composite Z‐score on the neurophysiologic test battery, and lower motor function score when compared to HC (uncorrected *p* < .05). However, the HIV + 12 wk shows no significant difference when compared to HC or to HIV + BSL. Cognitive performance improved in the HIV‐infected subjects after 12 weeks, as the boxplots show. The neuropsychological test scores in other domains, including speed, attention, learning, memory, executive function, and verbal fluency, although not significantly different between groups, were trending toward better performance in the HC which explain why the summary Z‐score was significantly different between HIV + BSL and HC. Neuropsychological test results for other cognitive domains that are not significantly different are reported in supplementary materials (Supplemental Figure S6).

We also investigated the relationship between Z‐scores for different cognitive domains and total Z‐score and global graph theoretical measurements. Figure 7c shows the significant linear relationship between neuropsychological test scores and graph theoretical measurements. The correlation coefficients are range from 0.19 to 0.25 with uncorrected *p* < .05.

## 4 DISCUSSION

In this study, we used rMFM whole‐brain dynamic modeling to investigate the local and global brain dynamic changes associated with HIV infection. Our results indicate that HIV infection disrupts microscale brain dynamics in several cortical regions, and HIV antiretroviral treatment improves these brain dynamics.

### 4.1 rMFM revealed disruption of local dynamics changes due to HIV infection

Among the areas found to have changes in both recurrent connection strength *w* and subcortical inputs *I* were the frontal poles, left lingual gyrus, and right lateral occipital areas. The frontal pole regions are part of the prefrontal cortex thus involved in working memory and multitasking (Gilbert et al., 2006). Disruption in these networks has significant behavioral consequences as shown by studies in HIV‐infected subjects with active cocaine use disorder (Meade, Lowen, MacLean, Key, & Lukas, 2011). Our study is also consistent with findings that HIV‐infection disrupts the frontostriatal circuitry (Heaton et al., 1995). The lingual gyrus (part of the visual processing system) has also been previously reported to be affected in perinatally HIV‐infected youths (Sarma et al., 2019). Ances et al. (2009) and Ances et al. (2010)) reported reduced activity and resting cerebral blood flow in the visual cortices during visual stimulation and at rest in HIV‐infected subjects using arterial spin labeling and fMRI. Wiesman et al. (2018)) also demonstrated that the spontaneous and neural oscillatory activity in the visual cortices were affected by HIV‐infection using magnetoencephalography. Our results are complementary to these observations, suggesting that the microscale cortical dynamics including recurrent connection strengths *w* and subcortical input strength *I* are affected in these areas in HIV infected individuals.

A number of regions, including left opercular part of inferior frontal gyrus (pars opercularis), superior temporal gyrus, postcentral gyrus, pericalcarine cortex, right insula, and parahippocampal gyrus showed significant differences only in recurrent connections strength *w*. Some of these, such as the insula and parahippocampal gyrus have been shown to have reduced regional volume and FC in the HIV‐infected population (Kallianpur et al., 2012; McIntosh et al., 2017; Samboju et al., 2018). The balanced integration of excitatory and inhibitory synaptic currents in the local cortex allows the cortical network to operate at high efficiency of information transmission at a low energy cost (Yu, Shen, Wang, & Yu, 2018). Although the rMFM model did not disentangle the contribution of excitatory and inhibitory effect, using rMFM method made it possible to compare local recurrent connection changes between groups.

Among the regions that showed significant differences only in subcortical inputs *I* were the left temporal pole, right temporal pole, right lingual gyrus, right isthmus cingulate, right precuneus, and right superior parietal regions. Some of these regions, such as temporal, right precuneus, superior parietal regions, have been reported to have altered in FC and amplitude of low frequency fluctuations in HIV infected subjects(Yadav et al., 2018). The medial frontal regions (bilateral frontal poles), precuneus, and isthmus cingulate gyrus are considered nodes within the default mode network (DMN). HIV‐associated decreases in FC within DMN are consistent with previously reports using ICA‐based FC analysis (du Plessis et al., 2017; Zhuang et al., 2017).

The bilateral supramarginal gyrus and inferior parietal lobule are considered as the two lateral hubs in the DMN. We found that the recurrent connection strength *w* of supramarginal gyrus was stronger in the HIV + BSL compared to HC, while the subcortical input strength *I* in the inferior parietal lobule was less in the HIV + BSL cohort compared to HC cohort. The significance of increased or decreased *w* and *I* are dependent on the local circuit. They are both average inputs that need to be compared to HC. Here, change with treatment helps to understand the meaning of the direction in the change observed. The DMN appears to be a critical network that is sensitive to HIV‐associated CNS injury.

We noticed the maximum similarity Z‐scores in HC group were consistently lower than the HIV + BSL and HIV + 12 wk groups, when using training dataset to optimize the rMFM model parameters. This difference indicates the rMFM model fitting is in favor of HIV‐infected group than HC group. We have tried to quantitatively compare the metastability of the three groups using the *SD* of the Kuramoto order parameter (Acebrón, Bonilla, Vicente, Ritort, & Spigler, 2005; Kuramoto, 1984; Strogatz, 2000). The metastability is not significantly different between groups. We also found the maximum similarity Z‐scores are comparable between groups, which indicates the rMFM models can produce a good and comparable representation of three groups.

In our study, we have shown that after treatment, the microscale local brain dynamics improved in both recurrent connection strength *w* and subcortical inputs *I*. Specifically, we found 9 out of 10 regions' rMFM metrics in HIV‐infected subjects change toward HC levels (Figures 3 and 4). A similar approach was reported by Deco et al. (Saenger et al., 2017) in the context of deep brain stimulation in Parkinson's disease, but to the best of our knowledge, our study is the first to use rMFM metrics to assess response in brain networks after cART.

### 4.2 Global and local topological organization disrupted in HIV‐infected subjects

Given that rMFM and simulated FC are population‐based analyses, we could not directly correlate individual subject cognitive scores and rMFM metrics *w* and *I*. We used an intermediate step by applying graph theoretical analysis to empirical FC and then correlated graph metrics to rMFM metrics. We found significant topological changes in FC when comparing HIV + BSL to HC, but no significant change in SC. The local topological measurements showed some association with the cognitive scores. Furthermore, we found after 12‐week cART treatment, the functional network topology in HIV‐infected subjects also shows transitions toward HC, similar to what was observed in local brain dynamics. Specifically, we observed that smallworldness, global clustering coefficient, and network efficiency were lower in the HIV + BSL (see Figure 5), which indicated the HIV‐infection changes the functional network integration and segregation. A previous study has shown that global clustering coefficient declines with HIV infection (Abidin, D'Souza, Nagarajan, & Wismuller, 2016). The loss of the smallworld organization in brain functional networks has been previously demonstrated in several neurological disorders, such as AD (Sanz‐Arigita et al., 2010), Parkinson's disease (Baggio et al., 2014), and traumatic brain injury (Pandit et al., 2013). In our study, we found that reduced smallworldness in HIV + BSL was associated with decreased neuropsychological composite Z‐score, executive function and learning Z‐score (Figure 7c).

The global network efficiency was also reduced in HIV + BSL, suggesting that the communication between different brain regions (van den Heuvel, Stam, Kahn, & Hulshoff Pol, 2009) was affected in HIV‐infected subjects. The global network efficiency was associated with the composite Z‐score, verbal, executive, and learning Z‐score (Figure 7c). The positive correlation of the network efficiency and executive function performance has been reported in HIV‐infected individuals (Ventura et al., 2018), where they focused on the posterior cingulate cortex.

We and others did not find significant topology difference in structural network of HIV infected individuals (Samboju et al., 2018; Zhuang et al., 2017). However, two studies (Baker et al., 2017; Bell et al., 2018) have shown disrupted structural networks in HIV‐infected subjects compared to HC in terms of reduced global clustering coefficient, global network efficiency, and connection strength. These two studies differ from our study in the population enrolled. Our patients had been recently diagnosed and had a relatively high CD4 count at baseline (mean 515.8 cells/mm^3^) compared to the other two studies. It is likely that persistence of HIV infection overtime causes some irreversible structural CNS changes (Zhu et al., 2013).

The local topological measurements showed some association with the local dynamic properties, specifically, we found the local topological measurements of FC were also correlated with the recurrent connection strengths *w* and subcortical inputs *I* (Figure 6 and Supplemental Figure S13). The positive correlations between recurrent connection strength *w* and clustering coefficient, and local efficiency, respectively, were only found in HC group, suggesting that HIV‐infection altered microscale local dynamics, which was also reflected in the change of network topology.

We found the association between microscale local dynamics and empirical network topology using both the Desikan atlas and Destrieux atlas. The HC group consistently shows higher associations than HIV + BSL and HIV + 12 wk (Figure 6a,b, and Supplemental Figure S13a,b). In HIV + 12 wk, a significant correlation was only present in Destrieux atlas. This may be due to the inclusion of more cortical parcels in the Destrieux atlas. The results suggest that the cART treatment tends to improve microscale brain dynamics and the efficiency of the brain information transmission.

### 4.3 Limitations

There are some limitations of our approach due in part to the available dataset. First, the rMFM modeling requires reliable and accurate SC reconstruction. Our diffusion data have only one shell at *b*‐value = 1,000 s/mm^2^. This single shell scheme may introduce errors for tractography due to fiber‐crossing issue (Sotiropoulos et al., 2013). In our study, we have adopted a reliable tractography and SC reconstruction pipeline (Zhang et al., 2018) to mitigate to some extent. Second, we could only derive a population‐based whole‐brain dynamic model using averaged FC and SC. The reliability of empirical FC is crucial to the rMFM simulation. A recent study from Patricio group (Donnelly‐Kehoe et al., 2019) suggests that in order to derive reliable subject‐specific brain dynamics, the total length of rsfMRI acquisition should be 20 min with TR = 2 s. They used both static FC matrices (Pearson correlation of BOLD signal) and dynamic‐FC (FCD) metrics to evaluate the simulation results, while in our analysis we only used static FC matrices. Though the FCD estimation requires longer rsfMRI scanning time compared to static FC, our 5 min rsfMRI scan may still limit the static FC reliability to generate subject‐specific FC. In this regard, we attempted to derive subject‐specific rMFM models using individual SC and FC maps; however, the variance of the similarities was much greater than that of the population‐based model. We thought this large intersubject variation for rMFM came from both individual differences and SC/FC noise. It is difficult to disentangle these two effects in our current data. However, we have shown that using group‐averaged population‐based rMFM modeling is much more stable across different cohorts, and we validated these findings using a different atlas. Thus, the rMFM model parameters (recurrent connection strength *w*, and subcortical input strength *I*) and the simulated FC can only be used in comparing groups but not individual parameters, such as CD4 count or cognitive scores. The HIV‐infected subjects in our study were mostly male; however, in the HC group the male and female participants were evenly distributed. Although in the rMFM modeling sex differences between groups were embedded in the final results, we cannot fully assess the possible effect of sex on *w* and *I*. Finally, our sample size is relatively small, constraining the stability of rMFM modeling as we needed to split the cohorts into training and test groups. Unfortunately, enrolling cART naïve patients is quite challenging. Future studies using more advanced diffusion scheme and longer rsfMRI acquisition, should provide more reliable subject‐specific rMFM model reconstruction.

### 4.4 Conclusion

We investigated the effect of HIV infection on the microscale local dynamics derived by whole‐brain computational modeling. We have identified several brain regions where recurrent connection strengths and subcortical inputs differ among HC, HIV+ untreated, and HIV+ after treatment. Treatment improved local brain dynamics, and this was also reflected in improved brain network topology and cognitive performance. However, short‐term treatment did not fully reverse CNS injury. Whether further diverge on CNS injury and cognitive performance occurs between HIV+ and HIV− over longer periods of time will need to be addressed in future studies. In this regard, whole‐brain dynamic modeling is a promising approach for assessing CNS injury progression and response to interventions.

## CONFLICT OF INTERESTS

The authors declare no conflict of interests.

## ETHICS STATEMENT

The study was reviewed and approved by the Institutional Review Board at the University of Rochester Medical Center and all subjects signed a written informed consent prior to undergoing study procedures. All subjects underwent a comprehensive clinical, laboratory (chemistry, hematology, and urine analysis), neurocognitive, and neuroimaging evaluation. HIV‐infected individuals were assessed before and 12 weeks after starting cART, while HIV‐uninfected controls were assessed only once at baseline. Subjects' demographics are listed in Table 1 in results session.

## Supporting information


**Appendix** **S1.** Supporting Information.

## Data Availability

The datasets from the current study are available from the corresponding author on reasonable request. The codes are available at https://github.com/yzhuang4/whole_brain_modeling. The codes for rMFM model used in the paper are adopted from https://github.com/ThomasYeoLab/CBIG/tree/master/stable_projects/fMRI_dynamics/Wang2018_MFMem.

## References

[hbm25207-bib-0001] Abidin, A. Z. , DSouza, A. M. , Nagarajan, M. B. , Wang, L. , Qiu, X. , Schifitto, G. , & Wismuller, A. (2018). Alteration of brain network topology in HIV‐associated neurocognitive disorder: A novel functional connectivity perspective. NeuroImage: Clinical, 17, 768–777. 10.1016/j.nicl.2017.11.025 29527484 PMC5842750

[hbm25207-bib-0002] Abidin, A. Z. , D'Souza, A. M. , Nagarajan, M. B. , & Wismuller, A. (2016). Investigating changes in brain network properties in HIV‐associated neurocognitive disease (HAND) using mutual connectivity analysis (MCA). Medical imaging 2016‐biomedical applications in molecular, structural, and functional imaging. Proceedings of SPIE—The International Society for Optical Engineering, 9788, 97881w 10.1117/12.2217317 PMC5697155 29170586

[hbm25207-bib-0003] Acebrón, J. A. , Bonilla, L. L. , Vicente, C. J. P. , Ritort, F. , & Spigler, R. (2005). The Kuramoto model: A simple paradigm for synchronization phenomena. Reviews of Modern Physics, 77(1), 137–185. 10.1103/RevModPhys.77.137

[hbm25207-bib-0004] Alderson, T. H. , Bokde, A. L. W. , Kelso, J. A. S. , Maguire, L. , Coyle, D. , & Alzheimer's Disease Neuroimaging Initiative . (2018). Metastable neural dynamics in Alzheimer's disease are disrupted by lesions to the structural connectome. NeuroImage, 183, 438–455. 10.1016/j.neuroimage.2018.08.033 30130642 PMC6374703

[hbm25207-bib-0005] Ances, B. M. , Sisti, D. , Vaida, F. , Liang, C. L. , Leontiev, O. , Perthen, J. E. , … Grp, H. (2009). Resting cerebral blood flow A potential biomarker of the effects of HIV in the brain. Neurology, 73(9), 702–708. 10.1212/WNL.0b013e3181b59a97 19720977 PMC2734291

[hbm25207-bib-0006] Ances, B. M. , Vaida, F. , Yeh, M. J. , Liang, C. L. , Buxton, R. B. , Letendre, S. , … Ctr, H. N. R. (2010). HIV infection and aging independently affect brain function as measured by functional magnetic resonance imaging. Journal of Infectious Diseases, 201(3), 336–340. 10.1086/649899 20047503 PMC2804778

[hbm25207-bib-0007] Antinori, A. , Arendt, G. , Becker, J. T. , Brew, B. J. , Byrd, D. A. , Cherner, M. , … Wojna, V. E. (2007). Updated research nosology for HIV‐associated neurocognitive disorders. Neurology, 69(18), 1789–1799. 10.1212/01.WNL.0000287431.88658.8b 17914061 PMC4472366

[hbm25207-bib-0008] Baggio, H. C. , Sala‐Llonch, R. , Segura, B. , Marti, M. J. , Valldeoriola, F. , Compta, Y. , … Junque, C. (2014). Functional brain networks and cognitive deficits in Parkinson's disease. Human Brain Mapping, 35(9), 4620–4634. 10.1002/hbm.22499 24639411 PMC6869398

[hbm25207-bib-0009] Baker, L. M. , Cooley, S. A. , Cabeen, R. P. , Laidlaw, D. H. , Joska, J. A. , Hoare, J. , … Paul, R. H. (2017). Topological organization of whole‐brain white matter in HIV infection. Brain Connectivity, 7(2), 115–122. 10.1089/brain.2016.0457 28076974 PMC5359681

[hbm25207-bib-0010] Bell, R. P. , Barnes, L. L. , Towe, S. L. , Chen, N. K. , Song, A. W. , & Meade, C. S. (2018). Structural connectome differences in HIV infection: Brain network segregation associated with nadir CD4 cell count. Journal of Neurovirology, 24(4), 454–463. 10.1007/s13365-018-0634-4 29687404 PMC6105458

[hbm25207-bib-0011] Buxton, R. B. , Wong, E. C. , & Frank, L. R. (1998). Dynamics of blood flow and oxygenation changes during brain activation: The balloon model. Magnetic Resonance in Medicine, 39(6), 855–864. 10.1002/mrm.1910390602 9621908

[hbm25207-bib-0012] Chang, L. , & Shukla, D. K. (2018). Imaging studies of the HIV‐infected brain In Handbook of Clinical Neurology (Vol. 152, pp. 229–264). Amsterdam, The Netherlands: Elsevier 10.1016/B978-0-444-63849-6.00018-9 29604980

[hbm25207-bib-0013] Chockanathan, U. , AM, D. S. , Abidin, A. Z. , Schifitto, G. , & Wismuller, A. (2018). Identification and functional characterization of HIV‐associated neurocognitive disorders with large‐scale Granger causality analysis on resting‐state functional MRI. Proceedings of SPIE—The International Society for Optical Engineering, 10575 10.1117/12.2293888 PMC6258079 30505063

[hbm25207-bib-0014] Deco, G. , Cruzat, J. , Cabral, J. , Knudsen, G. M. , Carhart‐Harris, R. L. , Whybrow, P. C. , … Kringelbach, M. L. (2018). Whole‐brain multimodal neuroimaging model using serotonin receptor maps explains non‐linear functional effects of LSD. Current Biology, 28(19), 3065–3074 e3066. 10.1016/j.cub.2018.07.083 30270185

[hbm25207-bib-0015] Deco, G. , & Kringelbach, M. L. (2014). Great expectations: Using whole‐brain computational connectomics for understanding neuropsychiatric disorders. Neuron, 84(5), 892–905. 10.1016/j.neuron.2014.08.034 25475184

[hbm25207-bib-0016] Deco, G. , Ponce‐Alvarez, A. , Hagmann, P. , Romani, G. L. , Mantini, D. , & Corbetta, M. (2014). How local excitation‐inhibition ratio impacts the whole brain dynamics. The Journal of Neuroscience, 34(23), 7886–7898. 10.1523/JNEUROSCI.5068-13.2014 24899711 PMC4044249

[hbm25207-bib-0017] Deco, G. , Ponce‐Alvarez, A. , Mantini, D. , Romani, G. L. , Hagmann, P. , & Corbetta, M. (2013). Resting‐state functional connectivity emerges from structurally and dynamically shaped slow linear fluctuations. The Journal of Neuroscience, 33(27), 11239–11252. 10.1523/JNEUROSCI.1091-13.2013 23825427 PMC3718368

[hbm25207-bib-0018] Demirtas, M. , Falcon, C. , Tucholka, A. , Gispert, J. D. , Molinuevo, J. L. , & Deco, G. (2017). A whole‐brain computational modeling approach to explain the alterations in resting‐state functional connectivity during progression of Alzheimer's disease. NeuroImage: Clinical, 16, 343–354. 10.1016/j.nicl.2017.08.006 28861336 PMC5568172

[hbm25207-bib-0019] Desikan, R. S. , Segonne, F. , Fischl, B. , Quinn, B. T. , Dickerson, B. C. , Blacker, D. , … Killiany, R. J. (2006). An automated labeling system for subdividing the human cerebral cortex on MRI scans into gyral based regions of interest. NeuroImage, 31(3), 968–980. 10.1016/j.neuroimage.2006.01.021 16530430

[hbm25207-bib-0020] Destrieux, C. , Fischl, B. , Dale, A. , & Halgren, E. (2010). Automatic parcellation of human cortical gyri and sulci using standard anatomical nomenclature. NeuroImage, 53(1), 1–15. 10.1016/j.neuroimage.2010.06.010 20547229 PMC2937159

[hbm25207-bib-0021] Donnelly‐Kehoe, P. , Saenger, V. M. , Lisofsky, N. , Kuhn, S. , Kringelbach, M. L. , Schwarzbach, J. , … Deco, G. (2019). Reliable local dynamics in the brain across sessions are revealed by whole‐brain modeling of resting state activity. Human Brain Mapping, 40(10), 2967–2980. 10.1002/hbm.24572 30882961 PMC6865451

[hbm25207-bib-0022] du Plessis, L. , Paul, R. H. , Hoare, J. , Stein, D. J. , Taylor, P. A. , Meintjes, E. M. , & Joska, J. A. (2017). Resting‐state functional magnetic resonance imaging in clade C HIV: Within‐group association with neurocognitive function. Journal of Neurovirology, 23(6), 875–885. 10.1007/s13365-017-0581-5 28971331 PMC5780332

[hbm25207-bib-0023] Fois, A. F. , & Brew, B. J. (2015). The potential of the CNS as a reservoir for HIV‐1 infection: Implications for HIV eradication. Current HIV/AIDS Reports, 12(2), 299–303. 10.1007/s11904-015-0257-9 25869939

[hbm25207-bib-0024] Friston, K. J. , Harrison, L. , & Penny, W. (2003). Dynamic causal modelling. NeuroImage, 19(4), 1273–1302. 10.1016/s1053-8119(03)00202-7 12948688

[hbm25207-bib-0025] Friston, K. J. , Mechelli, A. , Turner, R. , & Price, C. J. (2000). Nonlinear responses in fMRI: The Balloon model, Volterra kernels, and other hemodynamics. NeuroImage, 12(4), 466–477. 10.1006/nimg.2000.0630 10988040

[hbm25207-bib-0026] Gilbert, S. J. , Spengler, S. , Simons, J. S. , Steele, J. D. , Lawrie, S. M. , Frith, C. D. , & Burgess, P. W. (2006). Functional specialization within rostral prefrontal cortex (area 10): A meta‐analysis. Journal of Cognitive Neuroscience, 18(6), 932–948. 10.1162/jocn.2006.18.6.932 16839301

[hbm25207-bib-0027] Girard, G. , Whittingstall, K. , Deriche, R. , & Descoteaux, M. (2014). Towards quantitative connectivity analysis: Reducing tractography biases. NeuroImage, 98, 266–278. 10.1016/j.neuroimage.2014.04.074 24816531

[hbm25207-bib-0028] Griffanti, L. , Salimi‐Khorshidi, G. , Beckmann, C. F. , Auerbach, E. J. , Douaud, G. , Sexton, C. E. , … Smith, S. M. (2014). ICA‐based artefact removal and accelerated fMRI acquisition for improved resting state network imaging. NeuroImage, 95, 232–247. 10.1016/j.neuroimage.2014.03.034 24657355 PMC4154346

[hbm25207-bib-0029] Heaton, R. K. , Grant, I. , Butters, N. , White, D. A. , Kirson, D. , Atkinson, J. H. , … Wolfson, T. (1995). The HNRC 500—Neuropsychology of HIV infection at different disease stages. HIV neurobehavioral research center. Journal of the International Neuropsychological Society, 1(3), 231–251. 10.1017/s1355617700000230 9375218

[hbm25207-bib-0030] Honey, C. J. , Sporns, O. , Cammoun, L. , Gigandet, X. , Thiran, J. P. , Meuli, R. , & Hagmann, P. (2009). Predicting human resting‐state functional connectivity from structural connectivity. Proceedings of the National Academy of Sciences of the United States of America, 106(6), 2035–2040. 10.1073/pnas.0811168106 19188601 PMC2634800

[hbm25207-bib-0031] Jenkinson, M. (2003). Fast, automated, N‐dimensional phase‐unwrapping algorithm. Magnetic Resonance in Medicine, 49(1), 193–197. 10.1002/mrm.10354 12509838

[hbm25207-bib-0032] Jenkinson, M. , Beckmann, C. F. , Behrens, T. E. , Woolrich, M. W. , & Smith, S. M. (2012). Fsl. NeuroImage, 62(2), 782–790. 10.1016/j.neuroimage.2011.09.015 21979382

[hbm25207-bib-0033] Jirsa, V. K. , Sporns, O. , Breakspear, M. , Deco, G. , & McIntosh, A. R. (2010). Towards the virtual brain: Network modeling of the intact and the damaged brain. Archives Italiennes De Biologie, 148(3), 189–205. 21175008

[hbm25207-bib-0034] Kallianpur, K. J. , Kirk, G. R. , Sailasuta, N. , Valcour, V. , Shiramizu, B. , Nakamoto, B. K. , & Shikuma, C. (2012). Regional cortical thinning associated with detectable levels of HIV DNA. Cerebral Cortex, 22(9), 2065–2075. 10.1093/cercor/bhr285 22016479 PMC3412442

[hbm25207-bib-0035] Kuramoto, Y. (1984). Chemical oscillations, waves, and turbulence. New York, NY: Springer‐Verlag 10.1007/978-3-642-69689-3

[hbm25207-bib-0036] Maier‐Hein, K. H. , Neher, P. F. , Houde, J. C. , Cote, M. A. , Garyfallidis, E. , Zhong, J. , … Descoteaux, M. (2017). The challenge of mapping the human connectome based on diffusion tractography. Nature Communications, 8(1), 1349 10.1038/s41467-017-01285-x PMC5677006 29116093

[hbm25207-bib-0037] McIntosh, R. C. , Chow, D. C. , Lum, C. J. , Hidalgo, M. , Shikuma, C. M. , & Kallianpur, K. J. (2017). Reduced functional connectivity between ventromedial prefrontal cortex and insula relates to longer corrected QT interval in HIV plus and HIV‐ individuals. Clinical Neurophysiology, 128(10), 1839–1850. 10.1016/j.clinph.2017.07.398 28826014 PMC5612780

[hbm25207-bib-0038] Meade, C. S. , Lowen, S. B. , MacLean, R. R. , Key, M. D. , & Lukas, S. E. (2011). fMRI brain activation during a delay discounting task in HIV‐positive adults with and without cocaine dependence. Psychiatry Research‐Neuroimaging, 192(3), 167–175. 10.1016/j.pscychresns.2010.12.011 PMC3097316 21546221

[hbm25207-bib-0039] Mollink, J. , Smith, S. M. , Elliott, L. T. , Kleinnijenhuis, M. , Hiemstra, M. , Alfaro‐Almagro, F. , … Miller, K. L. (2019). The spatial correspondence and genetic influence of interhemispheric connectivity with white matter microstructure. Nature Neuroscience, 22(5), 809–819. 10.1038/s41593-019-0379-2 30988526 PMC6517273

[hbm25207-bib-0040] Nabha, L. , Duong, L. , & Timpone, J. (2013). HIV‐associated neurocognitive disorders: Perspective on management strategies. Drugs, 73(9), 893–905. 10.1007/s40265-013-0059-6 23733447 PMC3735343

[hbm25207-bib-0041] Pandit, A. S. , Expert, P. , Lambiotte, R. , Bonnelle, V. , Leech, R. , Turkheimer, F. E. , & Sharp, D. J. (2013). Traumatic brain injury impairs small‐world topology. Neurology, 80(20), 1826–1833. 10.1212/WNL.0b013e3182929f38 23596068 PMC3908350

[hbm25207-bib-0042] Proix, T. , Bartolomei, F. , Guye, M. , & Jirsa, V. K. (2017). Individual brain structure and modelling predict seizure propagation. Brain, 140(3), 641–654. 10.1093/brain/awx004 28364550 PMC5837328

[hbm25207-bib-0043] Rubinov, M. , & Sporns, O. (2010). Complex network measures of brain connectivity: Uses and interpretations. NeuroImage, 52(3), 1059–1069. 10.1016/j.neuroimage.2009.10.003 19819337

[hbm25207-bib-0044] Saenger, V. M. , Kahan, J. , Foltynie, T. , Friston, K. , Aziz, T. Z. , Green, A. L. , … Deco, G. (2017). Uncovering the underlying mechanisms and whole‐brain dynamics of deep brain stimulation for Parkinson's disease. Scientific Reports, 7, 9882 10.1038/s41598-017-10003-y 28851996 PMC5574998

[hbm25207-bib-0045] Salimi‐Khorshidi, G. , Douaud, G. , Beckmann, C. F. , Glasser, M. F. , Griffanti, L. , & Smith, S. M. (2014). Automatic denoising of functional MRI data: Combining independent component analysis and hierarchical fusion of classifiers. NeuroImage, 90, 449–468. 10.1016/j.neuroimage.2013.11.046 24389422 PMC4019210

[hbm25207-bib-0046] Samboju, V. , Philippi, C. L. , Chan, P. , Cobigo, Y. , Fletcher, J. L. K. , Robb, M. , … RV304 Protocol Teams . (2018). Structural and functional brain imaging in acute HIV. NeuroImage: Clinical, 20, 327–335. 10.1016/j.nicl.2018.07.024 30101063 PMC6082997

[hbm25207-bib-0047] Sanford, R. , Fellows, L. K. , Ances, B. M. , & Collins, D. L. (2018). Association of brain structure changes and cognitive function with combination antiretroviral therapy in HIV‐positive individuals. JAMA Neurology, 75(1), 72–79. 10.1001/jamaneurol.2017.3036 29131878 PMC5833491

[hbm25207-bib-0048] Sanz‐Arigita, E. J. , Schoonheim, M. M. , Damoiseaux, J. S. , Rombouts, S. A. R. B. , Maris, E. , Barkhof, F. , … Stam, C. J. (2010). Loss of 'small‐world' networks in Alzheimer's disease: Graph analysis of fMRI resting‐state functional connectivity. PLoS One, 5(11), e13788 10.1371/journal.pone.0013788 21072180 PMC2967467

[hbm25207-bib-0049] Sarma, M. K. , Keller, M. A. , Macey, P. M. , Michalik, D. E. , Hayes, J. , Nielsen‐Saines, K. , … Thomas, M. A. (2019). White matter microstructure among perinatally HIV‐infected youth: A diffusion tensor imaging study. Journal of Neurovirology, 25(3), 313–323. 10.1007/s13365-018-0714-5 30610741 PMC6609517

[hbm25207-bib-0050] Saylor, D. , Dickens, A. M. , Sacktor, N. , Haughey, N. , Slusher, B. , Pletnikov, M. , … McArthur, J. C. (2016). HIV‐associated neurocognitive disorder—Pathogenesis and prospects for treatment. Nature Reviews. Neurology, 12(5), 309 10.1038/nrneurol.2016.53 PMC5842923 27080521

[hbm25207-bib-0051] Smith, S. M. (2002). Fast robust automated brain extraction. Human Brain Mapping, 17(3), 143–155. 10.1002/hbm.10062 12391568 PMC6871816

[hbm25207-bib-0052] Sotiropoulos, S. N. , Jbabdi, S. , Xu, J. , Andersson, J. L. , Moeller, S. , Auerbach, E. J. , … The WU‐Minn Human Connectome Project Consortium . (2013). Advances in diffusion MRI acquisition and processing in the Human Connectome Project. NeuroImage, 80, 125–143. 10.1016/j.neuroimage.2013.05.057 23702418 PMC3720790

[hbm25207-bib-0053] Stebbins, G. T. , Smith, C. A. , Bartt, R. E. , Kessler, H. A. , Adeyemi, O. M. , Martin, E. , … Moseley, M. E. (2007). HIV‐associated alterations in normal‐appearing white matter: A voxel‐wise diffusion tensor imaging study. Journal of Acquired Immune Deficiency Syndromes, 46(5), 564–573. 10.1097/qai.0b013e318159d807 18193498

[hbm25207-bib-0054] Strogatz, S. H. (2000). From Kuramoto to Crawford: Exploring the onset of synchronization in populations of coupled oscillators. Physica D: Nonlinear Phenomena, 143(1–4), 1–20. 10.1016/s0167-2789(00)00094-4

[hbm25207-bib-0055] Surampudi, S. G. , Misra, J. , Deco, G. , Bapi, R. S. , Sharma, A. , & Roy, D. (2019). Resting state dynamics meets anatomical structure: Temporal multiple kernel learning (tMKL) model. NeuroImage, 184, 609–620. 10.1016/j.neuroimage.2018.09.054 30267857

[hbm25207-bib-0056] Thomas, J. B. , Brier, M. R. , Ortega, M. , Benzinger, T. L. , & Ances, B. M. (2015). Weighted brain networks in disease: Centrality and entropy in human immunodeficiency virus and aging. Neurobiology of Aging, 36(1), 401–412. 10.1016/j.neurobiolaging.2014.06.019 25034343 PMC4268260

[hbm25207-bib-0057] Underwood, J. , Cole, J. H. , Caan, M. , de Francesco, D. , Leech, R. , van Zoest, R. A. , … for the Comorbidity in Relation to AIDS (COBRA) Collaboration . (2017). Gray and white matter abnormalities in treated human immunodeficiency virus disease and their relationship to cognitive function. Clinical Infectious Diseases, 65(3), 422–432. 10.1093/cid/cix301 28387814 PMC5850629

[hbm25207-bib-0058] van den Heuvel, M. P. , Stam, C. J. , Kahn, R. S. , & Hulshoff Pol, H. E. (2009). Efficiency of functional brain networks and intellectual performance. The Journal of Neuroscience, 29(23), 7619–7624. 10.1523/JNEUROSCI.1443-09.2009 19515930 PMC6665421

[hbm25207-bib-0059] van Wijk, B. C. M. , Stam, C. J. , & Daffertshofer, A. (2010). Comparing brain networks of different size and connectivity density using graph theory. PLoS One, 5(10), e13701 10.1371/journal.pone.0013701 21060892 PMC2965659

[hbm25207-bib-0060] Ventura, N. , Douw, L. , Correa, D. G. , Netto, T. M. , Cabral, R. F. , Lopes, F. C. R. , & Gasparetto, E. L. (2018). Increased posterior cingulate cortex efficiency may predict cognitive impairment in asymptomatic HIV patients. The Neuroradiology Journal, 31(4), 372–378. 10.1177/1971400918782327 29895218 PMC6111431

[hbm25207-bib-0061] Wang, J. , Wang, X. , Xia, M. , Liao, X. , Evans, A. , & He, Y. (2015). GRETNA: A graph theoretical network analysis toolbox for imaging connectomics. Frontiers in Human Neuroscience, 9, 386 10.3389/fnhum.2015.00386 26175682 PMC4485071

[hbm25207-bib-0062] Wang, P. , Kong, R. , Kong, X. , Liegeois, R. , Orban, C. , Deco, G. , … Thomas Yeo, B. T. (2019). Inversion of a large‐scale circuit model reveals a cortical hierarchy in the dynamic resting human brain. Science Advances, 5(1), eaat7854 10.1126/sciadv.aat7854 30662942 PMC6326747

[hbm25207-bib-0063] Wiesman, A. I. , O'Neill, J. , Mills, M. S. , Robertson, K. R. , Fox, H. S. , Swindells, S. , & Wilson, T. W. (2018). Aberrant occipital dynamics differentiate HIV‐infected patients with and without cognitive impairment. Brain, 141, 1678–1690. 10.1093/brain/awy097 29672678 PMC5972635

[hbm25207-bib-0064] Woolrich, M. W. , Ripley, B. D. , Brady, M. , & Smith, S. M. (2001). Temporal autocorrelation in univariate linear modeling of FMRI data. NeuroImage, 14(6), 1370–1386. 10.1006/nimg.2001.0931 11707093

[hbm25207-bib-0065] Yadav, S. K. , Gupta, R. K. , Hashem, S. , Bhat, A. A. , Garg, R. K. , Venkatesh, V. , … Haris, M. (2018). Changes in resting‐state functional brain activity are associated with waning cognitive functions in HIV‐infected children. NeuroImage: Clinical, 20, 1204–1210. 10.1016/j.nicl.2018.10.028 30391858 PMC6224323

[hbm25207-bib-0066] Yu, L. , Shen, Z. , Wang, C. , & Yu, Y. (2018). Efficient coding and energy efficiency are promoted by balanced excitatory and inhibitory synaptic currents in neuronal network. Frontiers in Cellular Neuroscience, 12, 123 10.3389/fncel.2018.00123 29773979 PMC5943499

[hbm25207-bib-0067] Zhang, Z. , Descoteaux, M. , Zhang, J. , Girard, G. , Chamberland, M. , Dunson, D. , … Zhu, H. (2018). Mapping population‐based structural connectomes. NeuroImage, 172, 130–145. 10.1016/j.neuroimage.2017.12.064 29355769 PMC5910206

[hbm25207-bib-0068] Zhu, T. , Zhong, J. , Hu, R. , Tivarus, M. , Ekholm, S. , Harezlak, J. , … Schifitto, G. (2013). Patterns of white matter injury in HIV infection after partial immune reconstitution: A DTI tract‐based spatial statistics study. Journal of Neurovirology, 19(1), 10–23. 10.1007/s13365-012-0135-9 23179680 PMC3568248

[hbm25207-bib-0069] Zhuang, Y. , Qiu, X. , Wang, L. , Ma, Q. , Mapstone, M. , Luque, A. , … Schifitto, G. (2017). Combination antiretroviral therapy improves cognitive performance and functional connectivity in treatment‐naive HIV‐infected individuals. Journal of Neurovirology, 23(5), 704–712. 10.1007/s13365-017-0553-9 28791662 PMC5655604

