# Supplementary material for: Whole‐brain computational modeling reveals disruption of microscale brain dynamics in HIV infected individuals
Source: Hum Brain Mapp. 2020 Sep 17;42(1):95–109. doi: 10.1002/hbm.25207 (PMC7721235; doi:10.1002/hbm.25207)
Supplement: Supplementary file 1 — Appendix S1. Supporting Information. [file HBM-42-95-s001.docx]

Whole-brain computational modeling reveals disruption of microscale brain dynamics in HIV infected individuals

Yuchuan Zhuang ^a^, Zhengwu Zhang ^b,c^ , Madalina Tivarus ^d,c^ , Xing Qiu ^b^ , Jianhui Zhong ^d,e,*^ , Giovanni Schifitto ^f,d,*^

^a^ Department of Electrical and Computer Engineering, University of Rochester, 500 Computer Studies Building, Rochester, New York 14627, USA

^b^ Department of Biostatistics and Computational Biology, University of Rochester Medical Center, 265 Crittenden Boulevard, CU 420630, Rochester, New York 14642, USA

^c^ Department of Neuroscience, University of Rochester Medical Center, 601 Elmwood Avenue, KMRB G.9602, Rochester, New York 14642, USA

^d^ Department of Imaging Sciences, University of Rochester Medical Center, 601 Elmwood Ave, Rochester, New York 14642, USA

^e^ Department of Biomedical Engineering, University of Rochester, 201 Robert B. Goergen Hall, Rochester, New York 14627, USA

^f^ Department of Neurology, University of Rochester Medical Center, 601 Elmwood Avenue, Rochester, New York 14642, USA

*Corresponding authors: Dr. Jianhui Zhong, 601 Elmwood Ave, Rochester, University of Rochester Medical Center, New York 14642, USA, E-mail address: [Jianhui_Zhong@URMC.Rochester.edu](mailto:Jianhui_Zhong@URMC.Rochester.edu)

Dr. Giovanni Schifitto, 601 Elmwood Ave, Rochester, University of Rochester Medical Center, New York 14642, USA, E-mail address: [Giovanni_Schifitto@URMC.Rochester.edu](mailto:Giovanni_Schifitto@URMC.Rochester.edu)

# **Supplementary Methods**

## **Preprocessing of resting-state fMRI**

The preprocessing of fMRI using FEAT(Jenkinson et al., 2012) including head motion correction carried by MCFLIRT (Jenkinson et al., 2002) using rigid-body registration to the reference image; B0 inhomogeneity correction using boundary-based registration (BBR) with a fieldmap and registration to T1w image; slice timing correction; brain extraction using BET (Smith, 2002); spatial smoothing using a Gaussian kernel with full width at half maximum (FWHM) of 5 mm; and linear trend removal by applying a high-pass temporal filtering. The T1w image was warped to a 2mm MNI standard space using FNIRT, and coregistered with the rsfMRI using linear transformation, yielding a functional image resampled in 2mm MNI standard space. MELODIC option was switched on to prepare for FIX, an ICA-based noise removal.

## **Simulated FC using rMFM model**

The MFM model was derived from a detailed spiking brain model (Deco & Jirsa, 2012), where each brain region is modeled by a spiking attractor network, represented by two pools of neuron populations: a population of excitatory pyramidal neurons and a population of GABAergic inhibitory neurons. Within each brain region, the excitatory neurons and the inhibitory neurons are fully connected, the strength of this intra-region connection defined as recurrent connection strength $w$. The inter-region connections are weighted by the structural connectivity strength $C_{\mathrm{ij}}$ multiplied by a global scaling factor $G$. MFM uses the dynamic mean field reduction of each local spiking attractor (Wong & Wang, 2006), to simplify the mathematical description of the spiking model while fairly approximating the dynamics of the spiking network (Deco et al., 2013). The improved MFM, rMFM, relaxes the recurrent connection strength $w$ and subcortical inputs $I$ in the MFM model, so that each brain region has different values of recurrent connection strength $w_{i}$ and subcortical inputs $I_{i}$. The rMFM within each brain region can be modeled using a set of coupled nonlinear stochastic differential equations, Eq. (1-3):

|  | $\frac{dS_{i}\left( t \right)}{\mathrm{dt}}= -\frac{S_{i}}{{}_{s}}+\left( 1-S_{i} \right)H\left( x_{i} \right)+v_{i}\left( t \right)$ | (1) |
| --- | --- | --- |
|  | $H\left( x_{i} \right)=\frac{ax_{i}-b}{1-exp\left( -d\left( ax_{i}-b \right) \right)}$ | (2) |
|  | $x_{i}= w_{i}JS_{i}+GJ\sum_{j} C_{\mathrm{ij}}S_{j}+I_{i}$ | (3) |

where $S_{i}$, $H\left( x_{i} \right)$, and $x_{i}$ denote the average synaptic gating variable, the population firing rate, and the total input current at $i$-th brain region respectively, $C_{\mathrm{ij}}$ is the streamline counts connecting$i$-th brain region and $j$-th brain region, representing the anatomical connection strengths between brain areas $i$ and $j$, $w_{i}$ is the recurrent connection strength at $i$-th brain region and $I_{i}$ is the excitatory subcortical input at $i$-th brain region. Here the total input current at $i$-th brain region $x_{i}$ is driven by incoming local recurrent inputs within the same cortical area $w_{i}JS_{i}$ , long range inputs from all other cortical area $\mathrm{GJ}\sum_{j} C_{\mathrm{ij}}S_{j}$, and subcortical inputs $I_{i}$. The inter-areal connections are weighted by the structural connectivity strength $C_{\mathrm{ij}}$. Parameter values for the input output function $H\left( x_{i} \right)$ are $a$ = 270(VnC), $b$ =1008(Hz), and $d$ = 0.154(s); the kinetic parameters are γ = 0.641, ${}_{s}$ = 100ms; the synaptic couplings are $J$ = 0.2609(nA)$; v_{i}\left( t \right)$ is the uncorrelated standard Gaussian noise and is the noise amplitude. These values are derived from Deco et. al (Deco et al., 2013).

Simulated BOLD time series were derived from the simulated neuronal activities using the Ballon-Windkessel hemodynamic model (Friston et al., 2003; Friston et al., 2000), which describes BOLD signal as a function of changes in neuronal activity, cerebral blood flow, cerebral blood volume, and deoxyhemoglobin content, represented by Eq.(4-7). In brief, for the i-th brain region, neuronal activity $S_{i}$ causes an increase in a vasodilatory signal $z_{i}$, which results in an increase in inflow $f_{i}$, with concomitant changes in blood volume $v_{i}$ and deoxyhemoglobin content$q_{i}$.

|  | $\frac{\partial z_{i}\left( t \right)}{\partial t}=S_{i}-z_{i}- \left( f_{i}-1 \right)$ | (4) |
| --- | --- | --- |

|  | $\frac{\partial f_{i}\left( t \right)}{\partial t}=z_{i}$ | (5) |
| --- | --- | --- |

|  | $\tau\frac{\partial v_{i}\left( t \right)}{\partial t}= f_{i}- {v_{i}}^{1/\alpha}$ | (6) |
| --- | --- | --- |

|  | $\tau\frac{\partial q_{i}\left( t \right)}{\partial t}= \frac{f_{i}}{\rho}\left[ 1- \left( 1- \rho\right)^{1/{f_{i}}} \right]-q_{i}{v_{i}}^{1/{\alpha-1}}$ | (7) |
| --- | --- | --- |

where $=0.65$ $per s$ is the rate of signal decay, $=0.41$ $per s$ is the rate of flow-dependent elimination, $\tau=0.98$ is the hemodynamic transit time, $\rho=0.34$ is the resting oxygen extraction fraction and $\alpha=0.32$ is the Gubb’s exponent (Grubb et al., 1974). BOLD signal for each brain area was estimated by Eq. (8).

|  | $\mathrm{BOLD}_{i}=V_{0}\left[ k_{1}\left( 1-q_{i} \right)+ k_{2}\left( 1-{q_{i}}/{v_{i}} \right)+k_{3}\left( 1-v_{i} \right) \right]$ | (8) |
| --- | --- | --- |

where $V_{0}$ = 0.02 is the resting blood volume fraction, and $k_{1}$, $k_{2}$, and $k_{3}$are parameters dependent on field strength, calculated by following Eq.(9-11) below, revised from the Buxton model (Buxton et al., 1998) as in (Heinzle et al., 2016; Stephan et al., 2007).

|  | $k_{1}=4.3v_{0}E_{0}\mathrm{TE}$ | (9) |
| --- | --- | --- |
|  | $k_{2}=\varepsilon r_{0}E_{0}\mathrm{TE}$ | (10) |
|  | $k_{3}=1-\varepsilon$ | (11) |

where $v_{0}\cong28.265\cdot B_{0}$ is the frequency offset at the outer surface of magnetized vessels and depends linearly on the main magnetic field strength $B_{0}$, which is 3T in our case; $r_{0}\cong110 Hz$ in a 3T MR scanner, is the intravascular relaxation rate as a function of oxygen saturation; $\varepsilon={S_{I}}/{S_{E}}\cong0.47$ represents the ratio between intravascular and extravascular MR signal.

The simulated neuronal activity and BOLD time series for each brain region were numerically integrated using Euler’s method. The length of the simulated BOLD time series was 7 minutes, with the first 2 minutes discarded to allow model activity and simulated fMRI signal to stabilize (Schirner et al., 2018). The BOLD signal was down sampled to 2 second to match our empirical rsfMRI imaging protocol.

## **Graph theoretical measurements calculation**

Mean clustering coefficient: the clustering coefficient for each node implies network segregation (Rubinov & Sporns, 2010; Watts & Strogatz, 1998). The mean clustering coefficient is the average clustering coefficient across all nodes, which can be calculated with Eq.(12).

|  | $C= \frac{1}{n}\sum_{i\in N} C_{i}=\frac{1}{n}\sum_{i\in N} \frac{2t_{i}}{k_{i}\left( k_{i}-1 \right)}$ | (12) |
| --- | --- | --- |

where $C_{i}$ is the clustering coefficient of node i ($C_{i}$ = 0 for $k_{i}$ < 2), $k_{i}$ is the degree of node i meaning the number of links connected to the node, $t_{i}$ is the number of closed triangles.

Characteristic path length: the mean shortest path length between all pairs of brain regions is calculated with Eq.(13).

|  | $L= \frac{1}{n}\sum_{i\in N} L_{i}=\frac{1}{n}\sum_{i\in N} \frac{\sum_{i\in N,j\neq i} d_{\mathrm{ij}}}{n-1}$ | (13) |
| --- | --- | --- |

where $d_{\mathrm{ij}}$ is the shortest path length between nodes $i$ and $j$. $L_{i}$ is the average distance between node $i$ and all other nodes.

Global efficiency: efficiency between two nodes is the inverse of the shortest path length between these brain regions. Global efficiency (Latora & Marchiori, 2001; Rubinov & Sporns, 2010) of the network is the average efficiency for all node pairs, and is calculated with Eq. (14).

|  | $E= \frac{1}{n}\sum_{i\in N} E_{i}=\frac{1}{n}\sum_{i\in N} \frac{\sum_{i\in N,j\neq i} d_{\mathrm{ij}}^{-1}}{n-1}$ | (14) |
| --- | --- | --- |

where $E_{i}$ is the efficiency of node $i$.

Network smallworldness: when the network is highly clustered, while it has approximately the same characteristic path length as random networks (Rubinov & Sporns, 2010; Watts & Strogatz, 1998), it’s considered as a small-world network. And it can be characterized by a single measurement, network smallworldness (Humphries & Gurney, 2008), as calculated with Eq. (4).

|  | $S= \frac{\frac{C}{C_{\mathrm{rand}}}}{\frac{L}{L_{\mathrm{rand}}}}$ | (15) |
| --- | --- | --- |

where $C$ and $C_{\mathrm{rand}}$ are the clustering coefficients, and $L$ and $L_{\mathrm{rand}}$ are the characteristic path length of the respective tested network and a random network.

To further investigate the relationship between the microscale brain dynamics derived from rMFM model and the topological properties, we also calculated the nodal clustering coefficient $C_{i}$ in Eq (12), local efficiency $E_{i}$ in Eq (14) for the FC in the training dataset for the 68 nodes in Desikan atlas for each group (the results for Destrieux atlas are reported in supplementary materials).

# **Supplementary Results**

## **Age matching in training and test groups.**

Supplementary Table 1. Mean and standard deviation of age in training and test groups

|  | Training group (subject number) | Test group (subject number) |
| --- | --- | --- |
| Healthy Control (HC) | 37.73±13.31 (n=22) | 37.52±12.35 (n=21) |
| HIV+ Baseline (HIV+BSL) | 35.83±13.66 (n=23) | 35.74±12.09 (n=23) |
| HIV+ 12-week (HIV+12wk) | 36.63±13.34 (n=16) | 36.44±14.51 (n=16) |

- 1. **FC simulation results using rMFM**

Supplementary Fig. 1. rMFM optimization results**.** rMFM model parameter estimations for HC (red), HIV+BSL (blue), and HIV+12wk(green). A). the similarity values between simulated FC and empirical FC changes across 500 iterations. The model estimation is improved across 500 iterations for each cohort. Each dot represents the similarity value between simulated FC and empirical FC. The recurrent connection strength $w$ , and subcortical input strength I were updated after each iteration. B). the maximum similarity changed across 25 random initializations for each cohort. Each dot represents the maximum similarity value after 500 iterations; the rMFM model parameters corresponding to this maximum similarity value were recorded. For each cohort, we run the rMFM modeling using 25 different random initialization parameters, which yielded a total of 75 simulations. Red: HC, Blue: HIV+BSL, Green: HIV+12wk.

In Supplementary Fig. 1A, each dot represents the similarity value, calculated as the Pearson’s correlation between the simulated FC and empirical FC after each iteration. The similarity value increased dramatically (simulated FC converging to the empirical FC) in the first 100 iterations, and then plateaued after 250 iterations. Supplementary Fig. 1A shows that 500 iterations are sufficient for the purpose of model optimization. After 500 iterations were completed, we extracted the maximum similarity and its corresponding rMFM model parameters. We repeated this step with 25 different random initializations for each cohort, the maximum similarity Z-scores of each random initializations are shown in Supplementary Fig. 1B.

Supplementary Fig. 2. rMFM model parameters. rMFM model parameters across different randomized initialization parameters for each cohort, A). HC. B). HIV+BSL, C). HIV+12wk. Each plot is a 138x25 matrix. Each column represents the optimized rMFM model parameters after 500 iterations using 1 random initialization. We used 25 different random initializations for each cohort, yielding 25 columns in each plot. The first 68 rows indicate $w_{i}$ -recurrent connection strength for 68 ROIs, second 68 rows indicate $I_{i}$ – subcortical strength for 68 ROIs. The 137th row indicates the $G$-global scaler, and the 138th row indicates the $\sigma$ – amplitude of gaussian noise.

Supplementary Fig. 3. rMFM model validation results. rMFM model validations using test datasets for HC, HIV+BSL, and HIV+12wk. The three plots from top to bottom are similarity results for HC, HIV+BSL, and HIV+12wk respectively. The blue dots and line are the similarity results using training datasets across 1000 simulations, while the red dots and line are the similarity results using testing datasets across 1000 simulations.

Supplementary Fig. 3 indicates the similarity results of simulated FC and empirical FC through 1000 simulations using test dataset. The averaged similarity values for each cohort for the test datasets are 0.413, 0.414, and 0.433 respectively. For each cohort, the simulation results using the test dataset decreased 0.044-0.140 compared with simulation using training dataset, but still relatively high (above 0.41) indicating a good rMFM estimation.

- 1. **Local brain dynamic properties changes**

Supplementary Table 2. List of regions showed significant differences in w and I.

| Recurrent Connection strengths | | | |
| --- | --- | --- | --- |
| Hemisphere | Region Name | Normal Functions | Reported HIV-related defects |
| Left | Lingual Gyrus | Visual identification of facial expressions of emotions involved bilateral activation of the lingual gyrus (Kitada et al., 2010) | Regional increases in FA in the PHIV youth group compared to the HC group (Sarma et al., 2019) |
| Left | Pars Opercularis Gyrus | The pars opercularis refers to the vertically oriented fold of the inferior frontal gyrus in the frontal lobe of the brain, which plays a significant role in the production of speech (Petrides, 2014; Ulmer & Jansen, 2013) | Within the HIV+ group, the current CD4+ count was positively correlated with increased activity in the inferior frontal gyrus (Thames et al., 2016) |
| Left | Peri-calcarine Cortex | Associated with visual and sensory information-processing (Jalbrzikowski et al., 2013) | Blindsight subjects show significant increases in peri-calcarine cortical thickness (Georgy et al., 2020) |
| Left | Postcentral Gyrus | This region perceives various somatic sensations from the body, including touch, pressure, temperature, and pain (Lloyd et al., 2015) | Significant lower amplitude of low frequency fluctuations (ALFF) and FC in left postcentral gyrus in HIV-infected children compared to controls (Yadav et al., 2018)  MEG data showed significantly reduced theta responses in the postcentral gyrus in HIV-infected patients compared with controls (Wilson et al., 2015) |
| Left | Superior Temporal Gyrus | The superior temporal gyrus has been involved in the perception of emotion in facial stimuli, and is an essential structure involved in auditory processing and social cognition (Bigler et al., 2007; Jou et al., 2010; Radua et al., 2010) | Youths with autism have been shown to exhibit decreased gray matter volume, as well as anterior and superior displacement of the superior temporal gyrus, compared to typically developing controls (Stigler & McDougle, 2013)  Abnormalities of the superior temporal gyrus and associated language area have emerged as prominent biomarkers in schizophrenia research. Structural and functional deficits of these regions have been extensively examined in the context of auditory hallucinations (Meyer-Lindenberg et al., 2015) |
| Left | Frontal Pole Cortex | The frontal pole cortex contributes to many cognitive task, such as solving various puzzles, the Wisconsin card sorting task, and tasks involving inductive, analogical or relational reasoning, as well as prospective memory (Ramnani & Owen, 2004; Tsujimoto et al., 2011) | Cocaine dependence HIV-infected subjects show significantly smaller increases in activation than no lifetime substance dependence participants during hard versus easy choices task in the right frontal pole cortex (Meade et al., 2011) |
| Right | Lateral Occipital Cortex | The lateral occipital cortex is a mid-level visual region, and it’s particularly sensitive to object shape (Cant & Goodale, 2007; Emberson et al., 2017) | HIV-infected patients exhibit markedly increased functional MRI activation in lateral occipital during attentional processing.(Chang et al., 2001; Chang et al., 2004)  MEG study showed the spontaneous alpha and gamma activity in lateral occipital cortex is increase in HIV-infection and HAND (Wiesman et al., 2018) |
| Right | Parahippocampal Gyrus | The parahippocampal gyrus plays an important role in both spatial memory (Squire & Zola-Morgan, 1991) and navigation (Aguirre et al., 1996). | The ADC value was found significantly increased in Severe-impairment HIV-infected group compared to mild-impairment group. |
| Right | Frontal Pole Cortex | The frontal pole cortex contributes to many cognitive task, such as solving various puzzles, the Wisconsin card sorting task, and tasks involving inductive, analogical or relational reasoning, as well as prospective memory (Ramnani & Owen, 2004; Tsujimoto et al., 2011) | Cocaine dependence HIV-infected subjects show significantly smaller increases in activation than no lifetime substance dependence participants during hard versus easy choices task in the right frontal pole cortex (Meade et al., 2011) |
| Right | Insula | The insula contributes to multiple functions critical for human cognition and behavior. It involves sensorimotor processing, pain, socio-emotional processing, and high-level attention and decision making (Uddin et al., 2017) | The HIV-infected patients with lower CD4 counts and higher QTc intervals showed greater rsFC between the right VMPFC and the right posterior insula. (McIntosh et al., 2017)  There is a significant difference of regional homogeneity values in perinatal HIV-infected adolescents in Insula (Wang et al., 2018) |
| Subcortical Input strengths | | | |
| Left | Lingual | Visual identification of facial expressions of emotions involved bilateral activation of the lingual gyrus (Kitada et al., 2010) | Regional increases in FA in the PHIV youth group compared to the HC group (Sarma et al., 2019) |
| Left | Frontal Pole Cortex | The frontal pole cortex contributes to many cognitive task, such as solving various puzzles, the Wisconsin card sorting task, and tasks involving inductive, analogical or relational reasoning, as well as prospective memory (Ramnani & Owen, 2004; Tsujimoto et al., 2011) | Cocaine dependence HIV-infected subjects show significantly smaller increases in activation than no lifetime substance dependence participants during hard versus easy choices task in the right frontal pole cortex (Meade et al., 2011) |
| Left | Temporal pole | The temporal pole is involved with multimodal analysis, especially in social and emotional processing. The left temporal pole is associated with semantic memory (meanings, names, and general impersonal facts) (Snowden et al., 2004) | There is a significant decrease of ReHo when compared the perinatal HIV-infected adolescents to controls (Wang et al., 2018) |
| Right | Isthmus of Cingulate Gyrus | The isthmus of the cingulate, which connects the posterior cingulate cortex (PCC) to the parahippocampal gyrus, the function of which isn’t studied sufficiently (McLaren et al., 2016) | Depression is associated with changed volumes, thickness, and surface area in this region.(McLaren et al., 2016)  The cortical thickness of isthmus of the cingulate gyrus showed trended significant interaction of HIV diagnosis with methamphetamine dependence (MacDuffie et al., 2018) |
| Right | Lateral Occipital Cortex | The lateral occipital cortex is a mid-level visual region, and it’s particularly sensitive to object shape (Cant & Goodale, 2007; Emberson et al., 2017) | HIV-infected patients exhibit markedly increased functional MRI activation in lateral occipital during attentional processing.(Chang et al., 2001; Chang et al., 2004)  MEG study showed the spontaneous alpha and gamma activity in lateral occipital cortex is increase in HIV-infection and HAND (Wiesman et al., 2018) |
| Right | Lingual | Visual identification of facial expressions of emotions involved bilateral activation of the lingual gyrus (Kitada et al., 2010) | Regional increases in FA in the PHIV youth group compared to the HC group (Sarma et al., 2019) |
| Right | Precuneus | The precuneus involves in a variety of complex functions, which include recollection and memory, integration of information relating to perception of the environment, cue reactivity, mental imagery strategies, episodic memory retrieval, and affective responses to pain (Borsook et al., 2015) | A smaller volume of parietal precuneus was found with moderate to severe subjective neuropathy in HIV-infected patients.(Zahr et al., 2019)  A MRS study has found that, in the precuneus, the median NAA/Cr, and glutamate/Cr was significantly lower in the symptomatic HIV-associated neurocognitive disorder group (HAND) compared with asymptomatic neurocognitive impairment (ANI) group (Mohamed et al., 2018) |
| Right | Superior Parietal Lobule | The superior parietal lobule is involved in aspects of attention and visuospatial perception, including the representation and manipulation of objects (Johns, 2014) | An MEG study found that HIV-infected persons exhibited decreased beta oscillations in the superior parietal lobule relative to healthy controls.(Becker et al., 2013)  Decreased mean diffusivity (MD) was found in perinatally HIV-infected youths compared to healthy youths (Sarma et al., 2019) |
| Right | Frontal Pole Cortex | The frontal pole cortex contributes to many cognitive task, such as solving various puzzles, the Wisconsin card sorting task, and tasks involving inductive, analogical or relational reasoning, as well as prospective memory (Ramnani & Owen, 2004; Tsujimoto et al., 2011) | Cocaine dependence HIV-infected subjects show significantly smaller increases in activation than no lifetime substance dependence participants during hard versus easy choices task in the right frontal pole cortex (Meade et al., 2011) |
| Right | Temporal pole | The temporal pole is involved with multimodal analysis, especially in social and emotional processing. The right temporal pole is related to personal and episodic memories, being more closely associated with emotion and socially relevant memory (Nakamura et al., 2001) | There is a significant increase of ReHo when compared the perinatal HIV-infected adolescents to controls (Wang et al., 2018) |

Supplementary Fig. 4. Ranked differences of whole-brain rMFM model parameters, recurrent connection strengths $w$ (left), and subcortical inputs $I$ (right) for each brain region. First column: regional recurrent connection strength difference between HIV+BSL and HC, sorted by the difference (diff = HC – HIV+BSL). Second column: the difference of $w$, and $I$ were mapped on the brain surface, indicating the anatomical locations. Hotter color towards red means HC is greater than HIV+BSL, cooler color towards blue means HC is smaller than HIV+BSL. Third column: regional subcortical inputs difference between HIV+BSL and HC, sorted by the difference.

Supplementary Fig. 4 shows the difference of rMFM model parameters between HIV+BSL and HC, sorted by the difference (diff = HC – HIV+BSL) of recurrent connection strength $w$, and subcortical inputs $I$. The brain plots color-coded the difference between HC and HIV+BSL. The hotter regions indicate the rMFM model parameters are greater in HC, while the cooler regions indicate the values are greater in HIV. The highest differences of recurrent connections between HC and HIV+BSL were found in right frontal pole, right lateral occipital lobe, right parahippocampal gyrus, right insula, right supramarginal gyrus, and left supramarginal gyrus. The highest differences of subcortical input strength between HC and HIV+BSL were found in right inferio parietal lobule, left inferio parietal lobule, right fusiform, right lateral orbitofrontal, left isthmus of the cingulate gyrus, and left superioparietal lobule. These results also indicate that the HIV-infection altered the local cortical dynamics in the brain, so we only calculate the difference between HC and HIV+BSL in this analysis.

- 1. **rMFM model validation**

We further validated the rMFM estimation using a different training /test dataset for each cohort, and also run the model optimization with 500 and 1000 iterations, using 10~15 different initial parameters to further validate our model. Supplementary Fig. 5 A and B show the model optimization results after 500 iterations, Supplementary Fig. 5 C and Supplementary Fig. 5 D show the results after 1000 iterations. The plateau appears around 250 iterations for both cases, indicating 500 iterations of model parameter estimation is a good number to get a satisfactory and stable simulated FC. The maximum similarity values are in the range of 0.5 to 0.65, which were the same as previous results. Supplementary Fig. 5E and Supplementary Fig. 5F are calculated from the rMFM of validation test datasets with 500 iterations.

Supplementary Fig. 5. rMFM model validation. A) the similarity values between simulated FC and empirical FC changes across 500 iterations. B). the maximum similarity changed across different random initializations for each cohort. C) the similarity values between simulated FC and empirical FC changes across 1000 iterations. D). the maximum similarity changed across different random initializations for each cohort across 1000 iterations. E) rMFM models generated simulated FC for HC, HIV+BSL, and HIV+12wk, respectively. F) rMFM model validations. The three plots from top to bottom are similarity results for HC, HIV+BSL, and HIV+12wk respectively. The blue dots and line are the similarity results using training datasets across 1000 simulations, while the red dots and line are the similarity results using test datasets across 1000 simulations.

- 1. **Neuropsychological test score**

Supplementary Fig. 6 Neuropsychological test z-score for other cognitive domains: Speed of information process, attention, learning, memory, executive function, and verbal fluency Z-score. No significant difference was found between HIV+BSL and HC (uncorrected p>0.1).

- 1. **rMFM modeling results using Destriuex atlas**

We also replicated the rMFM model simulation using a finer parcellated atlas, the Destriuex atlas. The simulation results can be found in Supplementary Fig. 7. The rMFM simulation performed well on a finer segmented atlas. The similarities across 20 random initializations are within 0.54-0.61. The simulated FC and empirical FC in the test dataset also worked well, see Supplementary Fig. 5, yielding similarities are 0.439, 0.392, and 0.447 for HC, HIV+BSL, and HIV+12wk, respectively.

Supplementary Fig. 7. Validation the rMFM model parameter estimations using Destrieux atlas for HC (red), HIV+BSL (blue), and HIV+12wk(green). A). the similarity values between simulated FC and empirical FC changes across 500 iterations. The model estimation is improved across 500 iterations for each cohort. Each dot represents the similarity value between simulated FC and empirical FC. The recurrent connection strength $w$ , and subcortical input strength $I$ were updated after each iteration. B). the maximum similarity changed across 20 random initializations for each cohort. Each dot represents the maximum similarity value after 500 iterations; the rMFM model parameters corresponding to this maximum similarity value were recorded. For each cohort, we run the rMFM modeling using 20 different random initialization parameters, which yielded a total of 60 simulations. Red: HC, Blue: HIV+BSL, Green: HIV+12wk.

Supplementary Fig. 8. rMFM models generated the simulated FC for HC, HIV+BSL, and HIV+12wk, respectively using Destrieux atlas. Left column A) D) G): the averaged empirical FC in test dataset for each cohort. Middle column B) E) H): the averaged simulated FC. Right column C) F) I): The correlation between empirical and simulated FC.

Supplementary Fig. 9. rMFM model validations using Destrieux atlas, using test datasets. The three plots from top to bottom are similarity results for HC, HIV+BSL, and HIV+12wk respectively. The blue dots and line are the similarity results using training datasets across 1000 simulations, while the red dots and line are the similarity results using testing datasets across 1000 simulations.

Supplementary Fig. 10. The regional recurrent connection strengths results using Destrieux atlas. Top: the regional recurrent connection changes in these nodes plotted on a smoothed brain surface indicate their anatomical location. Bottom: bar plots show the recurrent connection strength for each ROIs. Red: HC, Blue: HIV+BSL, Green: HIV+12wk.

Supplementary Fig. 11. The subcortical input strengths results using Destrieux atlas. Top the subcortical inputs change in these nodes plotted on a smoothed brain surface indicate their anatomical location. Bottom: bar plots show the recurrent connection strength for each ROIs. Red: HC, Blue: HIV+BSL, Green: HIV+12wk.

Supplementary Fig. 12. Graph theoretical measurements on weighted functional connectivity matrix across network sparsity range from 0.05 to 0.5. A). smallworldness. B). global clustering coefficient. C). global characteristic path length. D). global network efficiency. * FDR corrected p<0.05. Red: HC, Blue: HIV+BSL, Green: HIV+12wk.

Supplementary Fig. 13. The recurrent connection $w$ and subcortical inputs$I$ correlated with empirical local network topology using Destrieux atlas. (A) Association between clustering coefficient and recurrent connection strength $w$. (B) Association between local efficiency and recurrent connection strength $w$. (C) Association between clustering coefficient and subcortical inputs $I$. (D) Association between local efficiency and subcortical inputs $I$. Red: HC, Blue: HIV+BSL, Green: HIV+12wk. ns: not significant

- 1. **Local brain dynamic properties changes when compare HC vs. HIV+BSL, HIV+BSL vs. HIV+12wk, separately.**

Reference:

Aguirre, G. K., Detre, J. A., Alsop, D. C., & D'Esposito, M. (1996, Nov-Dec). The parahippocampus subserves topographical learning in man. *Cereb Cortex, 6*(6), 823-829. <https://doi.org/10.1093/cercor/6.6.823>

Becker, K. M., Heinrichs-Graham, E., Fox, H. S., Robertson, K. R., Sandkovsky, U., O'Neill, J., Swindells, S., & Wilson, T. W. (2013, Dec). Decreased MEG beta oscillations in HIV-infected older adults during the resting state. *J Neurovirol, 19*(6), 586-594. <https://doi.org/10.1007/s13365-013-0220-8>

Bigler, E. D., Mortensen, S., Neeley, E. S., Ozonoff, S., Krasny, L., Johnson, M., Lu, J., Provencal, S. L., McMahon, W., & Lainhart, J. E. (2007). Superior temporal gyrus, language function, and autism. *Dev Neuropsychol, 31*(2), 217-238. <https://doi.org/10.1080/87565640701190841>

Borsook, D., Maleki, N., & Burstein, R. (2015). Migraine. In M. J. Zigmond, L. P. Rowland, & J. T. Coyle (Eds.), *Neurobiology of Brain Disorders* (pp. 693-708). Academic Press. <https://doi.org/10.1016/b978-0-12-398270-4.00042-2>

Buxton, R. B., Wong, E. C., & Frank, L. R. (1998, Jun). Dynamics of blood flow and oxygenation changes during brain activation: the balloon model. *Magnetic Resonance in Medicine, 39*(6), 855-864. <https://doi.org/10.1002/mrm.1910390602>

Cant, J. S., & Goodale, M. A. (2007, Mar). Attention to form or surface properties modulates different regions of human occipitotemporal cortex. *Cereb Cortex, 17*(3), 713-731. <https://doi.org/10.1093/cercor/bhk022>

Chang, L., Speck, O., Miller, E. N., Braun, J., Jovicich, J., Koch, C., Itti, L., & Ernst, T. (2001, Sep 25). Neural correlates of attention and working memory deficits in HIV patients. *Neurology, 57*(6), 1001-1007. <https://doi.org/10.1212/wnl.57.6.1001>

Chang, L., Tomasi, D., Yakupov, R., Lozar, C., Arnold, S., Caparelli, E., & Ernst, T. (2004, Aug). Adaptation of the attention network in human immunodeficiency virus brain injury. *Ann Neurol, 56*(2), 259-272. <https://doi.org/10.1002/ana.20190>

Deco, G., & Jirsa, V. K. (2012, Mar 7). Ongoing Cortical Activity at Rest: Criticality, Multistability, and Ghost Attractors. *Journal of Neuroscience, 32*(10), 3366-3375. <https://doi.org/10.1523/Jneurosci.2523-11.2012>

Deco, G., Ponce-Alvarez, A., Mantini, D., Romani, G. L., Hagmann, P., & Corbetta, M. (2013, Jul 3). Resting-state functional connectivity emerges from structurally and dynamically shaped slow linear fluctuations. *J Neurosci, 33*(27), 11239-11252. <https://doi.org/10.1523/JNEUROSCI.1091-13.2013>

Emberson, L. L., Crosswhite, S. L., Richards, J. E., & Aslin, R. N. (2017, Mar 29). The Lateral Occipital Cortex Is Selective for Object Shape, Not Texture/Color, at Six Months. *J Neurosci, 37*(13), 3698-3703. <https://doi.org/10.1523/JNEUROSCI.3300-16.2017>

Friston, K. J., Harrison, L., & Penny, W. (2003, Aug). Dynamic causal modelling. *Neuroimage, 19*(4), 1273-1302. <https://doi.org/10.1016/s1053-8119(03)00202-7>

Friston, K. J., Mechelli, A., Turner, R., & Price, C. J. (2000, Oct). Nonlinear responses in fMRI: the Balloon model, Volterra kernels, and other hemodynamics. *Neuroimage, 12*(4), 466-477. <https://doi.org/10.1006/nimg.2000.0630>

Georgy, L., Lewis, J. D., Bezgin, G., Diano, M., Celeghin, A., Evans, A. C., Tamietto, M., & Ptito, A. (2020, Jun). Changes in peri-calcarine cortical thickness in blindsight. *Neuropsychologia, 143*, 107463. <https://doi.org/10.1016/j.neuropsychologia.2020.107463>

Grubb, R. L., Jr., Raichle, M. E., Eichling, J. O., & Ter-Pogossian, M. M. (1974, Sep-Oct). The effects of changes in PaCO2 on cerebral blood volume, blood flow, and vascular mean transit time. *Stroke, 5*(5), 630-639. <https://doi.org/10.1161/01.str.5.5.630>

Heinzle, J., Koopmans, P. J., den Ouden, H. E. M., Raman, S., & Stephan, K. E. (2016, Jan 15). A hemodynamic model for layered BOLD signals. *Neuroimage, 125*, 556-570. <https://doi.org/10.1016/j.neuroimage.2015.10.025>

Humphries, M. D., & Gurney, K. (2008, Apr 30). Network 'small-world-ness': a quantitative method for determining canonical network equivalence. *PLoS One, 3*(4), e0002051. <https://doi.org/10.1371/journal.pone.0002051>

Jalbrzikowski, M., Jonas, R., Senturk, D., Patel, A., Chow, C., Green, M. F., & Bearden, C. E. (2013). Structural abnormalities in cortical volume, thickness, and surface area in 22q11.2 microdeletion syndrome: Relationship with psychotic symptoms. *Neuroimage Clin, 3*, 405-415. <https://doi.org/10.1016/j.nicl.2013.09.013>

Jenkinson, M., Bannister, P., Brady, M., & Smith, S. (2002, Oct). Improved optimization for the robust and accurate linear registration and motion correction of brain images. *Neuroimage, 17*(2), 825-841. <https://doi.org/10.1006/nimg.2002.1132>

Jenkinson, M., Beckmann, C. F., Behrens, T. E., Woolrich, M. W., & Smith, S. M. (2012, Aug 15). Fsl. *Neuroimage, 62*(2), 782-790. <https://doi.org/10.1016/j.neuroimage.2011.09.015>

Johns, P. (2014). Functional neuroanatomy. In P. Johns (Ed.), *Clinical Neuroscience* (pp. 27-47). Churchill Livingstone. <https://doi.org/10.1016/b978-0-443-10321-6.00003-5>

Jou, R. J., Minshew, N. J., Keshavan, M. S., Vitale, M. P., & Hardan, A. Y. (2010, Nov 11). Enlarged right superior temporal gyrus in children and adolescents with autism. *Brain Res, 1360*, 205-212. <https://doi.org/10.1016/j.brainres.2010.09.005>

Kitada, R., Johnsrude, I. S., Kochiyama, T., & Lederman, S. J. (2010, Jan 15). Brain networks involved in haptic and visual identification of facial expressions of emotion: an fMRI study. *Neuroimage, 49*(2), 1677-1689. <https://doi.org/10.1016/j.neuroimage.2009.09.014>

Latora, V., & Marchiori, M. (2001, Nov 5). Efficient behavior of small-world networks. *Phys Rev Lett, 87*(19), 198701. <https://doi.org/10.1103/PhysRevLett.87.198701>

Lloyd, D. M., McGlone, F. P., & Yosipovitch, G. (2015, May). Somatosensory pleasure circuit: from skin to brain and back. *Exp Dermatol, 24*(5), 321-324. <https://doi.org/10.1111/exd.12639>

MacDuffie, K. E., Brown, G. G., McKenna, B. S., Liu, T. T., Meloy, M. J., Tawa, B., Archibald, S., Fennema-Notestine, C., Atkinson, J. H., Jr., Ellis, R. J., Letendre, S. L., Hesselink, J. R., Cherner, M., Grant, I., & Group, T. (2018). Effects of HIV Infection, methamphetamine dependence and age on cortical thickness, area and volume. *Neuroimage Clin, 20*, 1044-1052. <https://doi.org/10.1016/j.nicl.2018.09.034>

McIntosh, R. C., Chow, D. C., Lum, C. J., Hidalgo, M., Shikuma, C. M., & Kallianpur, K. J. (2017, Oct). Reduced functional connectivity between ventromedial prefrontal cortex and insula relates to longer corrected QT interval in HIV+ and HIV- individuals. *Clin Neurophysiol, 128*(10), 1839-1850. <https://doi.org/10.1016/j.clinph.2017.07.398>

McLaren, M. E., Szymkowicz, S. M., O'Shea, A., Woods, A. J., Anton, S. D., & Dotson, V. M. (2016, Apr 19). Dimensions of depressive symptoms and cingulate volumes in older adults. *Transl Psychiatry, 6*(4), e788. <https://doi.org/10.1038/tp.2016.49>

Meade, C. S., Lowen, S. B., MacLean, R. R., Key, M. D., & Lukas, S. E. (2011, Jun 30). fMRI brain activation during a delay discounting task in HIV-positive adults with and without cocaine dependence. *Psychiatry Res, 192*(3), 167-175. <https://doi.org/10.1016/j.pscychresns.2010.12.011>

Meyer-Lindenberg, A., Tost, H., & Schwarz, E. (2015). Translational Medicine in Psychiatry. In M. Wehling (Ed.), *Principles of Translational Science in Medicine* (pp. 195-213). Academic Press. <https://doi.org/10.1016/b978-0-12-800687-0.00021-9>

Mohamed, M., Barker, P. B., Skolasky, R. L., & Sacktor, N. (2018, Apr). 7T Brain MRS in HIV Infection: Correlation with Cognitive Impairment and Performance on Neuropsychological Tests. *AJNR Am J Neuroradiol, 39*(4), 704-712. <https://doi.org/10.3174/ajnr.A5547>

Nakamura, K., Kawashima, R., Sugiura, M., Kato, T., Nakamura, A., Hatano, K., Nagumo, S., Kubota, K., Fukuda, H., Ito, K., & Kojima, S. (2001). Neural substrates for recognition of familiar voices: a PET study. *Neuropsychologia, 39*(10), 1047-1054. <https://doi.org/10.1016/s0028-3932(01)00037-9>

Petrides, M. (2014). *Neuroanatomy of language regions of the human brain*. Academic Press,. <http://www.sciencedirect.com/science/book/9780124055148> MIT Access Only

Radua, J., Phillips, M. L., Russell, T., Lawrence, N., Marshall, N., Kalidindi, S., El-Hage, W., McDonald, C., Giampietro, V., Brammer, M. J., David, A. S., & Surguladze, S. A. (2010, Jan 1). Neural response to specific components of fearful faces in healthy and schizophrenic adults. *Neuroimage, 49*(1), 939-946. <https://doi.org/10.1016/j.neuroimage.2009.08.030>

Ramnani, N., & Owen, A. M. (2004, Mar). Anterior prefrontal cortex: insights into function from anatomy and neuroimaging. *Nat Rev Neurosci, 5*(3), 184-194. <https://doi.org/10.1038/nrn1343>

Rubinov, M., & Sporns, O. (2010, Sep). Complex network measures of brain connectivity: uses and interpretations. *Neuroimage, 52*(3), 1059-1069. <https://doi.org/10.1016/j.neuroimage.2009.10.003>

Sarma, M. K., Keller, M. A., Macey, P. M., Michalik, D. E., Hayes, J., Nielsen-Saines, K., Deville, J., Church, J. A., Walot, I., & Albert Thomas, M. (2019, Jun). White matter microstructure among perinatally HIV-infected youth: a diffusion tensor imaging study. *J Neurovirol, 25*(3), 313-323. <https://doi.org/10.1007/s13365-018-0714-5>

Schirner, M., McIntosh, A. R., Jirsa, V., Deco, G., & Ritter, P. (2018, Jan 8). Inferring multi-scale neural mechanisms with brain network modelling. *Elife, 7*. <https://doi.org/10.7554/eLife.28927>

Smith, S. M. (2002, Nov). Fast robust automated brain extraction. *Hum Brain Mapp, 17*(3), 143-155. <https://doi.org/10.1002/hbm.10062>

Snowden, J. S., Thompson, J. C., & Neary, D. (2004, Apr). Knowledge of famous faces and names in semantic dementia. *Brain, 127*(Pt 4), 860-872. <https://doi.org/10.1093/brain/awh099>

Squire, L. R., & Zola-Morgan, S. (1991, Sep 20). The medial temporal lobe memory system. *Science, 253*(5026), 1380-1386. <https://doi.org/10.1126/science.1896849>

Stephan, K. E., Weiskopf, N., Drysdale, P. M., Robinson, P. A., & Friston, K. J. (2007, Nov 15). Comparing hemodynamic models with DCM. *Neuroimage, 38*(3), 387-401. <https://doi.org/10.1016/j.neuroimage.2007.07.040>

Stigler, K. A., & McDougle, C. J. (2013). Structural and Functional MRI Studies of Autism Spectrum Disorders. In J. D. Buxbaum & P. R. Hof (Eds.), *The Neuroscience of Autism Spectrum Disorders* (pp. 251-266). Academic Press. <https://doi.org/10.1016/b978-0-12-391924-3.00017-x>

Thames, A. D., Sayegh, P., Terashima, K., Foley, J. M., Cho, A., Arentoft, A., Hinkin, C. H., & Bookheimer, S. Y. (2016, Aug). Increased subcortical neural activity among HIV+ individuals during a lexical retrieval task. *Neurobiol Dis, 92*(Pt B), 175-182. <https://doi.org/10.1016/j.nbd.2015.10.017>

Tsujimoto, S., Genovesio, A., & Wise, S. P. (2011, Apr). Frontal pole cortex: encoding ends at the end of the endbrain. *Trends Cogn Sci, 15*(4), 169-176. <https://doi.org/10.1016/j.tics.2011.02.001>

Uddin, L. Q., Nomi, J. S., Hebert-Seropian, B., Ghaziri, J., & Boucher, O. (2017, Jul). Structure and Function of the Human Insula. *J Clin Neurophysiol, 34*(4), 300-306. <https://doi.org/10.1097/WNP.0000000000000377>

Ulmer, S., & Jansen, O. (2013). *fMRI basics and clinical applications* (2nd ed.). Springer,. SpringerLink <http://dx.doi.org/10.1007/978-3-642-34342-1> MIT Access Only

Wang, P., Li, J., Wang, X., Thapa, D., & Wu, G. Y. (2018, Aug). Asymptomatic Human Immunodeficiency Virus Vertical Transmitted Adolescents' Brain Functional Changes: Based on Resting-State Functional Magnetic Resonance Imaging. *AIDS Res Hum Retroviruses, 34*(8), 699-704. <https://doi.org/10.1089/AID.2017.0267>

Watts, D. J., & Strogatz, S. H. (1998, Jun 4). Collective dynamics of 'small-world' networks. *Nature, 393*(6684), 440-442. <https://doi.org/Doi> 10.1038/30918

Wiesman, A. I., O'Neill, J., Mills, M. S., Robertson, K. R., Fox, H. S., Swindells, S., & Wilson, T. W. (2018, Jun 1). Aberrant occipital dynamics differentiate HIV-infected patients with and without cognitive impairment. *Brain, 141*(6), 1678-1690. <https://doi.org/10.1093/brain/awy097>

Wilson, T. W., Heinrichs-Graham, E., Becker, K. M., Aloi, J., Robertson, K. R., Sandkovsky, U., White, M. L., O'Neill, J., Knott, N. L., Fox, H. S., & Swindells, S. (2015, Mar). Multimodal neuroimaging evidence of alterations in cortical structure and function in HIV-infected older adults. *Hum Brain Mapp, 36*(3), 897-910. <https://doi.org/10.1002/hbm.22674>

Wong, K. F., & Wang, X. J. (2006, Jan 25). A recurrent network mechanism of time integration in perceptual decisions. *J Neurosci, 26*(4), 1314-1328. <https://doi.org/10.1523/JNEUROSCI.3733-05.2006>

Yadav, S. K., Gupta, R. K., Hashem, S., Bhat, A. A., Garg, R. K., Venkatesh, V., Gupta, P. K., Singh, A. K., Chaturvedi, S., Ahmed, S. N., Azeem, M. W., & Haris, M. (2018). Changes in resting-state functional brain activity are associated with waning cognitive functions in HIV-infected children. *Neuroimage Clin, 20*, 1204-1210. <https://doi.org/10.1016/j.nicl.2018.10.028>

Zahr, N. M., Pohl, K. M., Pfefferbaum, A., & Sullivan, E. V. (2019, Sep). Dissociable Contributions of Precuneus and Cerebellum to Subjective and Objective Neuropathy in HIV. *J Neuroimmune Pharmacol, 14*(3), 436-447. <https://doi.org/10.1007/s11481-019-09837-2>
